# Report on the Progress of Human Anatomy and Physiology, in the Years 1843-4

**Published:** 1845-01

**Authors:** James Paget

**Affiliations:** Lecturer on General and Morbid Anatomy and Physiology, and Warden of the Collegiate Establishment, at St. Bartholomew's Hospital


PART THIRD.

### Original Reports and Memoirs.

### REPORT ON THE PROGRESS OF HUMAN ANATOMY AND PHYSIOLOGY IN THE YEARS 1843-4.

By James Paget,
Lecturer on General and Morbid Anatomy and Physiology, and Warden of the Collegiate Establishment, at St. Bartholomew's Hospital.

The purpose and the general plan of the following Report are similar to those of the last. The works noticed are, with a few exceptions, those published between the first day of October 1843, and the last of September 1844. It is, for many reasons, larger than that of last year; chiefly because more progress has been made in the sciences of which it treats, and many of the subjects in which that progress has been effected are too important to be lightly passed by, and too difficult for a brief account of them to be clear.

### CHEMICAL COMPOSITION OF THE BODY.

*Elementary constituents.* The researches of MM. Devergie and Hervy are known, which appeared to prove the existence of small quantities of copper and lead in the tissues of the human body. MM. Flandin and Danger, and the commission of the Academy of Medicine, contradicted these observations;* but M. Barse, in a paper, read before the Academy of Sciences of Paris, in August 1843, confirmed them by finding both the metals in the bodies of two persons to whom they could not have been given for poisons. The researches of Signor Cattanei di Momo seemed to prove that the metals do not exist in the bodies of new-born children or infants; and, now, M. J. Rossignon has given a probable account of the sources from which the bodies of adults derive one of them—the copper. He has found this metal in the blood and muscles of the dog, and in many articles of vegetable and animal food, e g. gelatine from bones, sorrel, chocolate, bread, coffee, succory, madder, and sugar. The ashes obtained from starch-sugar yield 4 per cent. of copper; those of gelatine 0·03 per cent.; those of bread 0 005 to 0·008 per cent.†

*Proteine compounds.* The most valuable contributions made this year to the science of animal chemistry are the works of Professor Mulder.‡ By his continued investigations he has discovered two new proteine-compounds of great importance, a binoxyde and a tritoxyde (bi-oxy- and tri-oxy-proteine). The former contains two, the latter three, equivalents more of oxygen than pure proteine does. One or both of these compounds exists ready-formed in arterial and all inflammatory blood: in the buffy coat; in pus; in false membrane; in vitelline

* Bulletin, Fevr. 19, 1839. † Medical Gazette, Dec. 1, 1843, from the Gazette Médicale.

‡ Proeve eener algemeene Physiologische Scheikunde; Rotterdam, 1843, 8vo. The part of this work which relates to all the proteine-compounds is translated by the writer in the Medical Gazette, Sept. and Oct. 1844; and a more complete account of the two compounds above mentioned is given in the same journal, Feb. 9, 1844, by Dr. G. Bird, from a paper in the Annalen der Chemie und Pharmacie, Bd. xlvii, p. 300.

substance; in cooked meat. It is probable that they are both formed by the oxydation of fibrine (or, at least, of that compound of proteine which appears as fibrine when blood coagulates), as often as the blood passes through the lungs. When blood coagulated by heat is digested with water, much of the tritoxyde of proteine is dissolved; and it appears to constitute a large portion of the *serosity, muco-extractive matter, and extrait de viande* of different authors. Mulder considers also that it is in its combination with proteine in these oxydes that oxygen is conveyed to the systemic capillaries (see page 253), where it is consumed, and whence, in place of the oxy-proteine, fibrine returns to the lungs in the venous blood.

Both compounds exist in excess in the blood during inflammatory diseases. They form the greater part of the buffy coat, and of false membrane. When a buffy coat is digested in water it is divided into two portions: one soluble, of which the greater part is hydrated tritoxyde of proteine, (and it is this which M. Bouchardat supposed to be gelatinous matter,) the other insoluble, consisting of binoxyde of proteine with fatty matter. The composition of false membranes is the same with the addition of gelatine. In pus the tritoxyde of proteine is what has been described under the name of pyine; a chief portion of vitelline substance is a sulphuret of the binoxyde.

The same compounds which are thus ready-formed in the living body may be, in several ways, artificially obtained. Both of them are formed by the oxydation of fibrine when it is boiled in water*; the tritoxyde alone is similarly formed from albumen. It is this tritoxyde which has been supposed to be gelatine obtained by the decomposition of albumen or fibrine when subjected to long boiling. The hydrated tritoxyde may be obtained by forming a chlorite of proteine (by passing a current of chlorine through a solution of albumen) and decomposing this with ammonia. The binoxyde may be obtained by the decomposition of the bisulphide of proteine which forms a chief constituent of hair. Lastly, when fibrine is partially dissolved in very dilute hydrochloric acid, the precipitate obtained by adding ammonia (the *albuminose* of Bouchardat) is an anhydrous tritoxyde of proteine; and the portion which is not dissolved by the acid is, probably, binoxyde of proteine. The formula of the binoxyde given by Mulder is C. 40, H. 62, N. 10, O. 14; that of the tritoxyde, C. 40, H. 62, N. 10, O. 15.†

M. Wurtz‡ finds in the products of the putrefaction of fibrine exposed for eight days in summer to the air, albumen, carbonic acid, acetic acid, *butyric acid* and ammonia. He considers that the production of the last-named acid indicates that fibrine (and, perhaps, the other proteine compounds) may be transformed into the neutral fatty bodies which are so intimately related to the volatile fatty acids.

He has also succeeded in removing from albumen of white of egg, all the inorganic matters through its combination with which it has been supposed to derive its solubility in water; and this without altering its solubility, or its other essential chemical characters.

*Gelatine.* Mulder's analyses from which he deduced his formula for gelatine (C. 13, H. 20, N. 4, O. 5), have been confirmed by Van Goudoever, who has also analysed gelatine which had lost its power of gelatinizing by long-continued boiling, and has found its composition to be C. 49·5, H. 6·56, N. 17·36, O. 26·58, or, in equivalents, C. 52, H. 82, N. 16, O. 21. He hence deduces that it is changed into a compound in which four equivalents of gelatine are united with one of water, (4 (C. 13, H. 20, N. 4, O. 5) + aq.); a compound analogous to one of those of gelatine with chlorous acid, discovered by Mulder (4 (C. 13, H. 20, N. 4, O. 5) × Cl. 2, O 3).§

* It is probably by a similar oxydation that we may explain the observation of Scherer, that moist fibrine when exposed to oxygen absorbs more of it than it gives off of carbonic acid.

† For these formulæ, the equivalents of the elements are considered to be O. 100, C. 76·437, H. 6·24, N. 88·36.

‡ Report of the Academie des Sciences, seances du 15 et 20 Avril, in the Gazette Médicale; and in the Ann. de Chimie et de Physique, Oct. 1844.

§ Physiologische Scheikunde, p. 351, where also is a suggestion of the mode in which gelatine may be formed from the proteine-compounds of the food.

*Fatty Principles.* Mulder has also discussed, at some length, the chemical physiology of the fatty matters of both plants and animals. He adopts the opinion of Berzelius and Redtenbacher* that the basis of neutral fats is an oxyde of a radical, which they name Lipyle, and of which the formula is C. 3, H. 4, that of the oxyde being C. 3, H. 4, O. With stearic, margaric, and elaic acids, this forms stearine, margarine, and elaine: and glycerine is not present (as such) in the fats, but is formed in their saponification by the union of two equivalents of the oxyde of lipyle, with three equivalents of water.

After suggesting the modes in which the several kinds of fat may be transformed in their passage from plants to animals, and from the body of one animal to another, he considers whether the neutral fats taken as food are conveyed, unaltered, into the tissues in which they are deposited, or are saponified, and again reduced into neutral fats by being made to combine with lipyl-oxyde in the body. He supposes they are saponified by the alkali of the bile and chyle; and that glycerine is, at the same time, formed, and carried with them into the blood. The question, in this case is, how is the neutral fat reproduced? for glycerine will not unite again with the fat-acids to form neutral fats. He supposes that the change is effected by the union of the fat-acids with nascent lipyl-oxyde, and that this is produced in such circumstances as, if more oxygen were present, might give rise to the formation of lactic acid. For when lactic acid is sublimed, a white substance is obtained which has the composition of a binoxyde of lipyl (C. 3, H. 4, O. 2), and of which two equivalents, when it is in contact with water, absorb one equivalent of water, and form lactic acid. And, for explanation of the way in which these changes may be brought about, he refers to the researches of Lehmann‡ on the influence of mixtures of fat and albumen in inducing chemical changes in other principles.§

*Spontaneous decomposition.* Dr. Helmholtz‖ has published some interesting experiments for the determination of the conflicting views respecting the nature of the so-called spontaneous decomposition of dead organic substances. The question has, for some time, been, whether these are due to the development of microscopic organisms, to which the substances decomposed serve as nutriment, and the secretions of which are the products of the decomposition; or whether the process is one of mere chemistry.

In the first place the author confirms the fact already known (but which Liebig tried to explain away) that these decompositions do not take place in substances which have been heated to the boiling point, and which are only supplied with air that has been exposed to a red heat. He found that azotized organic substances, derived from the animal proteine- and gelatine-compounds, remained unaltered for eight weeks in summer, though freely supplied with air, provided that air had all passed through a red-hot glass tube before coming to them. *They consumed the oxygen:* but no process of fermentation or decomposition could be detected: yet it soon commenced, with the attendant development of infusoria, when only a small quantity of air which had not been heated was introduced.

With other spontaneously-decomposing azotized compounds, these peculiarities are not observed. Hydrocyanic acid and urea decompose as rapidly at the boiling heat and in closed vessels, as they do when exposed to the air. Moreover, when urea decomposes in closed and heated vessels in which it is mixed with other organic substances, neither fermentation nor putrefaction is excited in them by its decomposition.

Again, organic substances do not ferment or putrefy when no oxygen is supplied to them except such as has been immediately derived from water decomposed by electricity. So that it appears that neither oxydation, nor the spontaneous decomposition (resembling putrefaction) of urea, nor the chemical movement pro-

* Annalen der Chemie und Pharmacie, August 1843.

† The formulæ given by Mulder are here adopted, in which water is considered as H. 2, O.

‡ In Simon's Beit. zur Phys. und Path. Chemie, Bd. i, p. 73.

§ Physiol. Scheikunde, p. 262-82.

‖ Müller's Archiv, 1843, Heft v.

duced by the electric current, nor the presence of any of those constituents of the atmosphere which remain undecomposed by a red heat, is capable of exciting the fermentation or putrefaction of organic substances. Since, however, the presence of some part of the atmosphere is necessary for it, it can be only by one or both of the two remaining constituents that it is excited, and these are, the exhalations of putrefying substances, and the germs of organic beings.

To determine which of these two is the real excitant of the process, Helmholtz separated them. He filled a vessel with fluid capable of putrefaction, but heated and excluded from the air, so that it would not putrefy spontaneously, and then he introduced into it another fluid, also capable of putrefaction, and which had not been heated nor excluded from the air; introducing it, however, by endosmosis through a bladder which even the smallest organic germs could not pass through. The fluid thus introduced excited putrefaction in that within the vessel, and the process went on as quickly as it would if the first fluid introduced had been exposed to the air. But its results were different from those of open putrefaction; the organic fluids though they developed gases and had a putrid smell, remained clear; the portions of flesh placed in them remained firm, though putrid; and no infusoria were produced. Experiments of the same kind did not succeed in exciting vinous fermentation. Although endosmosis took place, yet no gases were developed, and no yeast-vegetables were produced.

It appears then, 1st, That for putrefaction of animal substances (at least of the proteine- and gelatine-compounds), the most essential condition is the presence of putrefying particles of similar substances: though perhaps it may also be excited by a vital process. 2d. That the putrefaction of these substances differs from the spontaneous decomposition of other azotized compounds, by its capability of propagating itself. 3d. That it presents the most favorable condition for the development of living organisms; and, that this, though not essential to the process, modifies its result. And 4th. That the vinous fermentation is connected with the access of some solid body, which may be excluded by the interposition of a bladder, and which can only be supposed to be the *yeast-plant*. Of this yeast-plant, analyses confirming, though independent of, each other have been made by Mulder* and Schlossberger†. Carefully-purified yeast yields two distinct substances, of which one, comprising the cell-contents, and extracted by potash or acetic acid, resembles the proteine-compounds; and the other, comprising the cell-membranes, might be classed with the amylum or cellulose compounds.

As one of the products of spontaneous decomposition, Dr. Zimmerman‡ has illustrated the formation of the triple phosphate in numerous putrefying organic substances besides the urine. The crystals form much less abundantly, or not at all, when those compounds are removed from the several substances which, in their decomposition, may yield ammonia, such as proteine-compounds, mucus, pus, &c.

BLOOD.

*Coagulation*. An inexplicable case, in which the complete coagulation of the blood did not take place till fifteen days after its abstraction, has been published by Dr. Polli,§ whose former researches on the blood were given in the last Report,

* The analysis of Mulder is stated by Schlossberger to be in the first part of his Physiological Chemistry. This refers probably to the German translation, which was published later than the Dutch original, in which I find no such analysis.

† Annalen der Chemie und Pharmacie, August, 1844.

‡ Casper's Wochenschrift, Oct. 21, 1843.

§ Gazzetta Medica di Milano, Gennaio 20, 1844—*On the blood-corpuscles:* the discussion between Dr. Carpenter and Mr. Wharton Jones, whether it be by the red or by the pale corpuscles that the albumen is to be supposed to be transformed into fibrine, is continued in the three last preceding Nos. of this Review. I must be content to refer the reader to their several papers; and as to Mr. Addison's work 'On the Actual Process of Nutrition in the Living Structures,' and to his papers in the Prov. Med. and Surg. Trans. for 1843, and in several recent numbers of the Medical Gazette, for a further account of his views and observations respecting the apparent transformations of the pale corpuscles of the blood into pus-corpuscles, mucus-corpuscles, tissues, &c.

and who adduces this case in proof that there is no blood which becomes putrid before it has coagulated. The patient was a man 37 years old, with acute pneumonia. The blood of a first bleeding was drawn into a block-tin vessel, and set in a temperature between 46° and 52°. It remained liquid for eight days, the blood-corpuscles having settled to the bottom, with the liquor sanguinis floating above them, exactly like serum pressed from a clot. On the ninth day, a membranous pellicle began to form on the surface of the fluid, and this becoming thicker, and increasing in tenacity and consistence, acquired at last all the characters of the most genuine buffy coat. The serum began to ooze from the clot on the fifteenth day: and on the clot being now taken out of the vessel, it was found that the upper four-fifths of it consisted of buffy coat, and only the lower fifth of clot coloured with corpuscles. The serum which continued to be expressed was perfectly transparent, and the blood did not show signs of putrefaction till a month after it had been drawn from the body. (A small quantity drawn from another pneumonic patient, and placed under the same circumstances, was completely coagulated in two hours, and was quite putrid in fifteen days.) In fifteen bleedings of the same patient in the following eight days, the blood drawn gradually lost its indisposition to coagulate, the whole process being completed each time in twelve hours or less. The patient recovered.

*Colour.* Some experiments by Scherer* both confirm the opinion of Nasse,† that the change from the arterial to the venous colour of the blood depends in great measure on the form of the blood-corpuscles, and explain most of the observations of Dr. Stevens on the effects of distilled water and salts upon the blood. Their general conclusions are: 1. That when fresh-stirred and bright-red ox-blood is mixed with distilled water, it acquires a dark-red colour, and its corpuscles, by imbibing water, become spherical, and at last vanish. But, 2. That if, after the change has begun, and not gone far, a concentrated solution of a neutral salt be added, the blood-corpuscles again acquire their natural form, and the bright-red colour is restored. 3. That when oxygen is passed through blood darkened by the addition of distilled water, it is not changed in colour, and the blood-corpuscles do not reappear; but that the same kind of blood, mixed with a small quantity of milk, or oil, or finely-powdered chalk, or gypsum, soon regains its bright red colour. 4. Again, by the long-continued contact of concentrated saline solutions with the blood-corpuscles they become jagged and decomposed, and the blood becomes black; and those which have been reddened by the action of salts, become black again on being expanded by the imbibition of water. 5. By adding carbonic acid to bright-red blood, its corpuscles change their biconcave for a biconvex form, and at the same time its colour changes from red to black. So that there are always changes in the shape of the blood-corpuscles, coincident with the changes in the colour of the mass of blood; whenever they are dilated, as by distilled water or carbonic acid, the dark colour is produced; whenever they are contracted into the biconcave form, the bright-red colour is restored.

Mulder, also,‡ espouses the opinion of the changes of colour in the blood being immediately due to physical rather than to chemical changes of the corpuscles, and has added many facts to those just quoted in disproof of the opinion of Liebig, that the changes are due to the alternate production of the carbonate of the protoxyde, and of the peroxyde, of iron in the blood-corpuscles, as they pass alternately through the systemic and the pulmonary capillaries. His chief facts are—1. That the elementary composition of the colouring matter is the same, whether obtained from arterial or from venous blood, viz., C. 44, H. 44, N. 6, O. 6, Fe. 2. That the change from dark to bright blood is effected as completely by the agency of a neutral salt as by oxygen. 3. That if the iron were present in the blood as an oxyde (and especially as a peroxyde), it should be easily extracted by weak acids;

* Henle and Pfeuffer, Zeitschrift, &c., and Oesterr. Medic. Wochenschrift, Nov. 4, 1843.

† Handworterbuch der Physiologie; Art. Blut.

‡ Verslag van de Vertiende Vergadering van het Nederlandsche Instituut in 'Het Instituut,' 1844, No. iv, and Physiologische Scheikunde, pp. 361-77.

but he has found that well-prepared hæmatine may be digested in diluted hydrochloric or sulphuric acid for several days without the iron in it being in the least diminished. After being so treated he has obtained, after incineration, the regular proportion of 9·49 per cent. of oxyde.* If strong sulphuric acid be poured on dried blood, or dried pure hæmatine, and kept on it for some days, and then water be added, hydrogen is evolved, and sulphate of peroxyde of iron is found in the solution, which could not happen if the iron had been at first in the form of peroxyde.† 5. The iron may thus be all extracted from the blood, or from hæmatine, (though not, as some say, without affecting the colour,) and the other constituents may be obtained in a separate form. Numerous analyses of this constituent, by Van Goudoever, regularly yielded the same equivalents of the elements, viz., C. 44, H. 44, N. 6, O. 6; but if the iron had been united with this in the form of Fe. 2, O. 3, and in the proportion of one equivalent to two, there should have remained only four and a half equivalents of oxygen.

Mulder concludes, therefore, that iron is present in hæmatine, as iodine is in sponge, or sulphur in cystine, or arsenic in cacodyl. His notion of the mode in which the changes of colour are effected is, that when the corpuscles of the venous blood are exposed in the lungs, oxy-proteine is formed by the oxydation of the fibrine proteine of the liquor sanguinis, or, perhaps, by the oxydation of the outer layer of the cell membrane of the corpuscles. If formed in the liquor sanguinis, its peculiar plasticity would lead to its being deposited in a thin layer on the corpuscles. In either such case, the dark corpuscles would, after respiration, be invested by a thin layer of white and imperfectly transparent oxy-proteine, or buffy coat, through which they would look bright red, as dark blood does when contained in a vessel of milk-white glass. But, in the systemic capillaries, the oxy-proteine may be consumed in nutrition, and the darkness of the corpuscles will then again appear unveiled.

Moreover, since it appears that, in the biconcave form, the corpuscles by reflecting more light, are always bright, and in the biconvex form always dark, it may be that in the arterial blood they are not only *buffed*, but also *cupped*, by the oxy-proteine, [by the plastic properties of which, moreover, it is easy, on this pretty theory, to explain the ready adhesion of the corpuscles in inflammatory blood.] Diluted acids, which make bright blood dark, may do so by making the outer layer of the corpuscles transparent, as they do fibrine before dissolving it; and concentrated solutions of neutral salts may make it bright by making the same layer contract.

*Chemical composition.* M. Figuier‡ has suggested an easy method for the rough analysis of the blood. By adding to one volume of defibrinated blood, two volumes of a solution of sulphate of soda, of sp. gr. marking 16° to 18° in Baume's areometer, the corpuscles will separate (as Berzelius showed), and may, with hardly an exception, be all collected on a filter. Thus their quantity may be estimated, as that of the fibrine may [very roughly] by what is obtained by whipping. The quantity of albumen may be estimated by boiling the serum; and the water, by evaporating a separate portion of blood.

*Ashes.* Enderlin§ has carefully analysed, in Liebig's laboratory, the ashes of

* Liebig adduces the possibility of extracting iron from dried blood as one of the proofs of its being in an oxydized state; but Mulder says this iron must have been extracted from some other constituent of the blood; for others, besides the globules, even pure serum, contain iron.

† When the blood or its colouring matter has been exposed to the air or prepared in it, the iron must always, according to Liebig's view, be in the state in which he supposes it to be in arterial blood.

‡ Report of the Académie des Sciences du 8 Juillet 1844; and, in full, in the Ann. de Chimie et de Physique, Août, 1844.

§ Annalen der Chemie und Pharmacie, Marz und April, 1844. The same paper contains analyses of the blood-ashes of the calf, ox, sheep, and hare, confirming the above conclusions, and numerous miscellaneous observations on the chemical characters of the proteine-compounds. Other analyses from these papers are reported under the head of Saliva and Fæces. They contain also an analysis of the ashes of ox-flesh, which the author finds identical (in quality) with those of blood, confirming thereby the analyses of Playfair and Böckmann, who found a similar and even closer identity of composition between the complete blood and flesh.

the blood. Their solution in hot water formed a very alkaline fluid, which, in all cases, contained alkaline phosphates and sulphates, chloride of sodium, and, sometimes, chlorides of potassium. But, from various tests [which I have repeated, and found exactly true], he proves that—1. The alkaline reaction of the ashes cannot be due to an alkaline carbonate, for both the ashes and the precipitates from their solution by nitrate of silver and chloride of calcium, may be dissolved in acids without the development of gas. 2. The alkaline reaction cannot depend on the presence of caustic alkali; for then the solution could not be, as it is, neutral after the addition of a solution of neutral chloride of calcium. 3. The absence of alkaline carbonates and of carbonate of lime in the ashes of the blood, proves that its albumen cannot be in the form of a salt (albuminate) of soda; and furnishes additional evidence that there are no alkaline salts of lactic, acetic, or fatty acids in the healthy blood ; and, lastly, proves that the blood can contain no alkaline carbonate. 4. The alkaline character of the blood-ashes and of the blood itself, must therefore be due to the phosphate of soda; and the presence of the tribasic phosphate of soda in the ashes proves, according to Enderlin, that it must be in the same form (3 NaO., P. 2, O. 5—that of the basic phosphate of soda of earlier chemists,) in the blood itself; for this salt alone remains tribasic after a red heat—the common phosphate of soda would yield pyrophosphate after incineration. He shows also that this view of the alkaline nature of the blood is consistent with the phenomena of respiration, and all other facts: especially, solutions of both the basic phosphates of soda are distinguished, as the serum is, by readiness to absorb large quantities of carbonic acid.

The quantitative analysis of the ashes showed that, in 100 parts from human blood, there are:

| | |
|---|---|
| Tribasic phosphate of soda | 22·1 |
| Chloride of sodium | 54·769 |
| ,, potassium | 4·416 |
| Sulphate of soda | 2·461 |
| Phosphate of lime | 3·636 |
| ,, magnesia | 0·769 |
| Oxide of iron, with some phosphate of iron | 10·77 |

It follows from these analyses that the albumen in the blood is not in the form of an albuminate of soda, nor of a combination with carbonate or bicarbonate of soda, but is in combination with the alkaline tribasic phosphate and chloride. The former salt possesses, in a high degree, the power of dissolving proteine-compounds and phosphates of lime; and it is probable, therefore, that it is the solvent of both these constituents of the blood.

*Milky serum.* Dr. A. Buchanan,* by experimental bleedings, has confirmed the fact of the frequent or general occurrence of milky or opaque serum in the blood of healthy persons, after taking food. The serum, he says, becomes turbid about half an hour after taking food: the discoloration increases during several hours, attains its maximum in about six or eight hours (after a full meal by a healthy person), and then becomes gradually clearer, till its limpidity is restored. The opaque serum is generally milk-white, sometimes cream-yellow, or yellowish-brown, like thin oatmeal gruel; or it merely loses its limpidity, and is like weak syrup. It always contains solid white granules, smaller than the blood-corpuscles (spherical or irregular in form) which are suspended in it, and which will rise in a white cream to the surface, either spontaneously or after the fluid has been saturated with common salt. The cream thus obtained is soluble in caustic potash, but insoluble in ether and alcohol; and is considered by Dr. R. D. Thomson as probably a proteine-compound.

* Transactions of the Glasgow Philosophical Society, March, 1844; Extract in the London and Edinburgh Monthly Journal of Medical Science, July, 1844; and in the Medical Gazette, Oct. 4, 1844. The examinations are confirmatory of Mr. Gulliver's, (Gerber's Anatomy, Appendix, p. 22;) and of the general opinion, that the opacity of the serum is due to the admixture of chyle.

GENERAL ANATOMY AND PHYSIOLOGY OF THE TISSUES.

*Tendinous tissues.** S. Pappenheim† has described the nerves of several of these tissues. In the periosteum, whether covering the shafts or the articular ends of bones, he finds them very numerous, lying especially in the outer surface of the membrane, in company with or upon the arteries, and having terminal loops. In the ligaments, there are nerves, which, after ramifying in the cellular tissues covering them, penetrate with the processes of that cellular tissue, in company with or upon the vessels, into the substance of the ligaments and end in them with plexuses and loops. Nerves may be found in like manner in all capsular ligaments: and it may be expressed, as a general rule, that all ligaments which receive vessels receive also a small number of nerves, though it is but one or two primitive fibres. In tendons, nerves can only sometimes be traced: and Pappenheim has never traced them into human tendons. He supposes that here also the rule of nerves coexisting with arteries may hold; [but here he is wrong, for arteries may be injected in the toughest tendons;] he believes that he has proved the existence of both sympathetic, sensitive, and motor fibres, in all nerves of the fibrous tissues which he has yet examined.

*Serous membranes.* Reichert‡ describes (what Henle doubted) an epithelium on the interior of the tendinous and sub-cutaneous bursæ, like that lining arteries and the true serous membranes. He has also§ explained the error of his and Remak's observation of a supposed layer of cells within the epithelium of the blood-vessels.‖ The appearance is due to the formation of artificial vesicles by the action of water. It is often produced on the surface of serous membranes, gland-ducts, &c.; and is always likely to lead to error.

Valentin¶ has related some interesting experiments on the properties of animal membranes as *filters.* A solution [or suspension] of albumen so diluted that it appeared a homogeneous fluid under the microscope, when filtered through some horses' pleura previously dried, was separated into a more diluted fluid which passed through, and a more concentrated one which was retained upon the filter. A similar division was similarly effected in pure serum which had been repeatedly filtered through paper. Saline solutions passed through unaltered. [Probably it may be added that different serous membranes filter with different degrees of fineness, and that on this depends the differences of the fluids found in them after death. The fluid of the cerebral ventricles, for example, though having no characters of a true secretion, is peculiar; and, unlike the fluids of other serous membranes, is not tinged when the serum is coloured by madder, and is very rarely discoloured in jaundice. It appears to be a fluid more finely-filtered from the liquor sanguinis. Again, under the increased pressure from congestion, whether passive or active, the filtration of the fluid through the blood-vessels and serous membranes will be less fine: hence the general occurrence of soft jelly-like masses and thin strings of fibrine in the fluid effused in ascites from extreme obstruction of the circulation: hence, also, a probable explanation of what Bischoff** has remarked, that the abdominal cavity in rabbits and bitches at the time of heat often contains some pellucid fluid which almost all coagulates when left at rest. These facts also coincide with the observations of Mr. Robinson†† on the effects of obstruction of the renal circulation in producing effusion of parts of the blood into the urinary tubules: and with some of his recent illustrations‡‡ of the general effects of the increased lateral pressure of the blood on the walls of the minute vessels].§§

* On the Structure of the Fibro-cellular tissue, see Reichert's observations on the description of the nerve-fibres.

† Müller's Archiv, 1843, Heft v. ‡ Ib. 1844. Jahresbericht, p. 229. § Ib. p. 289.

‖ See Report, 1842, p. 39. ¶ Lehrbuch der Physiologie des Menschen, Bd. i, p. 601.

** Müller's Archiv, 1844, Jahresbericht, p. 120.

†† Medico-Chirurgical Trans., vol. xxvi, p. 51. See also the similar observations of Dr. H. Mayer, on the Effects of Congestion of the Vessels of the Digestive Canal, in Schmidt's Jahrbucher, Mai 1844.

‡‡ Medical Gazette, June 28, 1844.

§§ The observations included in brackets and most of those in the notes are the author's. See, further, Krause's account of Epidermis, with that of the Skin.

*Cuticles,* Mucus, &c.* Reichert,† from his own and Bidder's observations, denies that the epithelium is ever so shed from the digestive canal, in or after any act of digestion, as to leave any portion of the subjacent mucous membrane uncovered or *raw.* When it has appeared so, the epithelium which remained has probably been washed off after death. In connexion with the same subject are some experiments by Oesterlen‡ which have proved the influence of the layer of mucus which lines the digestive canal, in retarding both the imbibition of fluids inclosed within the canal and their permeation by endosmosis. The passage of fluid into or through the mucous membrane of the intestines was, in many cases, more than twice as rapid when the mucus had been removed as while it was yet adherent.

Mr. Quekett§ has detected a double movement of the ciliæ on the gill-rays of the mussel. Besides their commonly observed curved or lashing movement in a vertical plane, each row presents a slight movement of the ciliæ on themselves, each cilia turning on its own axis through the space of a quarter of a circle, with a movement like that of the *feathering* of an oar in rowing. It is almost certain that without a movement of this kind, it would not be possible for any ciliæ to propel fluid in any determinate direction, or to propel, as they do, separate particles, such as epithelium cells, to which they are attached.

Against the opinion of the transformation of young epithelium-cells into pus-corpuscles in inflammations, Dr. Bühlmann‖ states that in coryza and bronchial catarrh, the discharge of very numerous exudation-corpuscles takes place without ever being preceded by the separation of an unusual quantity of epithelium. Neither can there be found, in the first stages of these diseases, any of those bodies which are supposed to be intermediate between epithelium-cells and pus-corpuscles; nothing more than fully-developed epithelium-cells are to be found scattered in the abundant quantity of exudation-corpuscles; and these latter, as the disease becomes chronic, without any important change in the quantity or character of the cast-off epithelium, gradually passing through the intermediate stage of *mucus-corpuscles,* assume the characters of pus-corpuscles.

*Bones.*¶ Dr. Daubeny** has established the truth of the much-doubted†† opinion of Morichini and Berzelius, that fluorine, in the form of fluoride of calcium, is contained in recent as well as fossil bones and teeth. It appears to exist in recent bones in about a quarter of the proportion in which it exists in fossil bones; but the proportions in different specimens of both kinds are variable. The Professor ascribes the failure of those who have not detected the fluorine, except in fossil bones and teeth, to the tenacity with which it is retained by animal matter, and to its being carried off with the carbonic acid evolved at the same time too rapidly to act upon the glass submitted to it. He therefore, before submitting the bones to the action of strong sulphuric acid, burns away all the animal matters, removes the carbonic acid by dissolving them in hydrochloric acid, then throws down the earthy phosphates by caustic ammonia, and dries them.

The experiments have also been fully confirmed and extended by Mr. Middleton,‡‡ who has also detected fluorine in several kinds of deposits from water, and ascribes its comparative abundance in fossil bones (as MM. Gerardin and Preisser did††) to the filtration of water impregnated with it.

*Muscles, structure of.* Dr. F. R. Will§§ maintains that the transverse striæ of the animal muscular fibres are due to the fibrils (which, in their natural relaxed state, are, he believes, uniform and cylindrical,) being thrown, in contraction, into undulations or zig-zag flexures. His arguments for the natural cylindrical form of the filaments are, chiefly, the usual straightness of the longitudinal lines between

* See, further, Krause's account of Epidermis with that of the Skin.
† Müller's Archiv, 1844, Jahresbericht, p. 121.
‡ Beiträge zur Physiologie des gesunden und kranken Organismus, Jena, 1843, p, 241, e. s.
§ Medical Gazette, May 3, 1844.
‖ Beit. zur Kennt. der kr. Schleimhaut des Respirationsorgane; Bern. 1843, p. 40.
¶ On the Development of Bone, see the section on Development. ** Philosoph. Magazine, Aug. 1844.
†† See last Report. ‡‡ Philosoph. Mag. July and Oct. 1844. §§ Müller's Arch. 1843, Heft iv.

the fibrils, whether contracted or relaxed; the occasional appearance of unmarked fibrils protruding from the end of a torn fibre; and the frequency of uniform cylindrical filaments in muscle which has been long macerated. And his arguments for the zig-zag condition, or the undulation, to which he ascribes the appearance of transverse striæ, more or less contracted on the fibrils, are the following:

1. When the primitive fibres of the muscles recently taken from dying insects, contract under water, the transverse striæ, which at first were wide apart, are approximated; and in every such contraction, the clear as well as the dark transverse lines become narrower, the elevations at the margin of the fibre become more prominent, and the constrictions between them become deeper and narrower. This could not happen if the contraction depended on the formation and widening with flattening of beads, or varicose enlargements of the filaments; for, in this case, the shadows (dark transverse lines), thrown by the beads or enlarged parts, should become broader and more intense as the beads become larger; whereas, in increasing contraction they become more intense and narrower, as the zig-zag angles become more acute.

2. The longitudinal striæ separating primitive filaments, remain straight even when the filaments are most contracted; whereas if the filaments become beaded, these lines should indicate that form, in the same manner as it is seen at the edge of the fibre where side views of the zig-zag filaments are obtained.

3. In repeated observations of the muscles of crabs, some of which were partially dried, and others macerated in water, it was found that, in the former, the individual filaments which projected from the torn ends of fibres were, at their extremities, bordered by two perfectly straight lines, which, as they were traced down towards the mass of the fibre, became slightly undulated, then zig-zag in obtuse angles, and then in more and more acute angles, till they could not be recognized as continuous lines. In the macerated muscles, on the contrary, there was scarcely any trace of transverse striæ, and the filaments appeared in almost every part bounded by straight lines.

4. The appearance of transverse striæ, and of rows of spheres may be imitated by thin cylinders of white wax, or a similar substance, moulded in undulations or zig-zag flexures, and examined through a tube as they are held against the light, the angles next the observer appearing darker and broader than those next the light; and the same appearance is produced by the coarsely-undulated surface of many muscles and tendinous tissues.

[This last argument is of little weight; and the facts stated in the first and second are just as well explained by supposing, not that the fibrils present a series of varicose bead-like enlargements, but that they are formed by a series of discs, the lines of union of which make the dark transverse striæ; or, as Mr. Erasmus Wilson* has better expressed it, by a linear series of minute cells, flattened at their apposed surfaces, and so compressed longitudinally as to leave no indentation on the surface, thus constituting a uniform cylinder divided by transverse septa, which are formed by the adherent surfaces of contiguous cells. Will's third argument deserves attention; but as the observations were made on dried and macerated fibrils, no conclusion can be safely drawn till they are confirmed by observations on muscles in the natural state.]

Valentin,† who has long described the relaxed muscular fibril as a uniform cylinder, confirms, generally, Will's account, though he cannot determine whether the striated appearance of the fibrils is due to their becoming varicose, or to zig-zag flexures, induced by contraction. He also still holds to his belief ‡

* Report of the Royal Society, Philos. Magazine, Aug. 1844. What I have quoted from the description expresses almost exactly what has *appeared* to me to be the structure of the muscular fibril, especially in the large fibres of the eel; except that I doubt greatly whether the component portions of the fibril can be truly called *cells;* they have, rather, the appearance of solid transparent particles. Neither have I ever seen anything to indicate a varying density of the contents in each successive set of four cells such as Mr. Wilson describes: perhaps this appearance was due to the fibres or fibrils being thrown into the coarse zig-zag flexures, described by some as produced in full contraction of the muscle.

† Lehrbuch der Physiologie des Menschen, Bd. ii, p. 33.

‡ See Report for 1842, p. 30.

that the fibres and fasciculi in the fully-contracted state are inflected in zig-zag lines, with angles of from 80° to 120°. He mentions, for demonstration of this opinion, his observations on the exposed laryngeal muscles of the frog. His account is almost precisely like that of Hales,* by whom the zig-zag arrangement of fibres was first observed in the abdominal muscles of the frog; and in further evidence of this arrangement being assumed in contractions, he adds, that as often as it is seen (and it may take place seventy-four times in a minute) the small arteries and nerves between the fibres become tortuous (through the approximation of their extremities), and straighten themselves when the contraction ceases.

*Muscles. Force of contraction.* Valentin† has also described a very ingenious *myodynamometer* for testing the force of contraction in the muscles of frogs and other small animals; the force being estimated, not, as in Schwann's experiments by weights in a scale, but by the tension of a bow-spring. From numerous experiments with it he has deduced (besides confirmations of the results of Schwann), 1st. That when, after death, all the irritability has ceased, the muscular fibres tear with a far less weight, that they were previously able, when galvanized, to draw. 2. That by too frequent and rapid irritation the irritability of the muscles is so exhausted, that it is for a time reduced to zero; but that it collects again, though, to a less degree, the longer the animal has been dead, and the oftener its irritability has been exhausted. 3. That by repeated equal irritations, the strength of the muscles (in beheaded frogs) decreases in a regular and corresponding ratio, losing the same amount in each successive period of time. 4. That after tying the femoral artery or vein, or dividing the sciatic nerve (in frogs) the full strength of the muscles remains unaltered for several (in one case as many as twelve) days.

*Rigor mortis.* Dr. Gierlichs‡, of Bonn, has confirmed what I stated in the last Report (p. 6), respecting the rigor mortis of the heart, and the general rule of the rigor mortis setting in as soon as a muscle has ceased to be irritable by stimuli. In frogs, in which the rigor is often not established till three or four days after apparent death, the hind-legs do not become rigid till from six to twelve hours later than the fore-legs; and they were often irritable to galvanism when the fore-legs were already quite rigid. Various means which exhaust muscular irritability, such as poisoning by strychnia or hydrocyanic acid, accelerate the accession of the rigidity. After injections of potash into the blood, the rigor takes place with unusual rapidity, and is very marked. This affords an additional evidence against the notion of the rigidity being dependent on coagulation of the blood or effused liquor sanguinis, for the alkali would retard that process; and a corroborative fact is, that if blood be repeatedly drawn from a dog, and each portion that is abstracted be injected again after the removal of its fibrine, the rigor mortis takes place as usual. No destruction of nerves appears to affect it, unless the muscles have been so long paralytic that their nutrition has been affected. When one of the crural arteries of a frog was tied, the rigor mortis ensued several hours later in the corresponding limb than in the other. Its occurrence bears no apparent relation to the loss of heat.

Valentin,§ also, considers that the rigor mortis may be assumed to affect the involuntary muscles, and relates an experiment to prove its occurrence in the digestive canal. If a portion of intestine from an animal just slain be tied close at one end, and at the other be tied round a graduated tube, and be filled with water, some of which also rises into the tube, it will, after some hours, slowly contract, and, pressing the water from its cavity, will elevate considerably that which is in the tube.

* Statical Essays, vol. ii, p. 59.

† Physiologie, Bd. ii, pp. 176-92. See, in the section on nerves, the application of similar experiments by Matteucci to determine the force exerted by nerves in exciting muscular contraction.

‡ Medic-Correspondenz-blatt Rheinischer Aerzte, 1843, and Schmidt's Jahrbucher, Mai 1844.

§ Physiologie, Bd. ii, p. 86.

CIRCULATION.

*Anatomy and Physiology of the Heart.* Kürschner's article in the last published part of Wagner's Handworterbuch* contains all the results of his latest investigation of the actions and anatomy of the heart. The parts which are of most interest from their novelty are briefly as follows: 1. The contraction of an auricle begins at the entrance of the great veins and thence proceeds to the base of the auricle. This is evident, if the auricle, while acting, be looked at *from the side.* 2. The contraction of a ventricle begins simultaneously in every part, the whole cavity draws up uniformly to the origin of the artery: this has hitherto been overlooked because such large hearts have been usually examined, that it was impossible to take in the whole ventricle at a glance; but it is evident when hearts as small as a rabbit's are examined while acting slowly. 3. The auricles never completely empty themselves in their contraction.† 4. If fluid be injected into either side of the heart through any of the great veins, (the other veins being tied and the apex of the heart suspended loosely in its natural position), the apex is always moved backwards as the cavities become fuller. 5. If the injection be made through one of the left pulmonary veins, the apex of the heart, at the same time that it is moved backwards, rotates from left to right; if the injection be made through any of the other veins, the rotation is from right to left. 6. When after filling the cavities, the fluid is drawn back by raising the piston, the heart returns to its previous position, being made to pass through exactly contrary movements, apparently by the elasticity of the tissues disturbed in the previous distension. 7. It is probable, therefore, that the alternate filling and emptying of the heart's cavities during life, which these injections are intended to imitate, contributes materially to its natural movements of receding from the wall of the chest and rotating its apex from right to left in the dilatation of the ventricles, and of tilting forwards and rotating its apex from left to right in their contraction. 8. In the auriculo-ventricular valves [to give only such a general description as may apply to both] one may observe besides their two or three chief divisions or lobes, the arrangement of which is always the same, as many smaller and less regular ***intermediate lobes*** connecting the adjacent edges of the larger ones. Each chief lobe may be divided into a thicker middle or ***nucleus-portion,*** and a surrounding thinner and dentated ***marginal portion.*** 9. The number of fleshy columns projecting from the ventricular wall is always equal to the number of chief divisions of the valve. The tendinous cords attached to the outer surface of each chief division are always derived from two fleshy columns (or in the right ventricle from one column and the septum): the cords attached to each intermediate division or lobe of a valve are all derived from one column. Consequently on each column there are, usually, three groups of tendinous cords, of which the two outer groups belong to the halves of two adjacent chief divisions of the valve, and the middle one to a smaller intermediate division. 10. The tendinous cords may be divided into three classes: namely, ***tendons of the first order,*** of which two or four, of considerable size, and from different columns, go to the attached margin of each division of the valve, and are there fixed to the muscular tissue of the ventricle; ***tendons of the second order,*** which are of smaller size, and of which two or three are given from each column (directly or through the medium of one of the first order) to each chief

* A part of the contents of this article [*Herzthatigkeit*] was published before the last year, but not in a connected form, and they are scarcely at all known in England. The description of the valves and their cords is singularly accurate, though, if I may so speak, too diagram-like; and the arrangements are so nearly constant, that they must be of prime importance in securing the due action of the valves. The article contains, also, a good account of all the points discussed concerning the heart's action; and an ingenious theory of it by Kürschner, which is omitted here only because there is not space even for all the facts that should be noticed.

† [The ventricles probably *do* empty themselves. If a heart, in which the contraction of the rigor mortis is very marked, be divided transversely, it will often be found that the opposite inner walls of the left ventricles are in contact, and its cavity completely obliterated; indeed, the form and position of the fleshy columns appear to be specially arranged for this end.]

division. They are attached to the outer (or back) surface of the valve in such position that their points of attachment lie in straight lines, which run parallel or slightly converging, from the attachments of the tendons of the first order towards the apex of their division of the valve. Both these kinds of tendons may be seen on looking at the back of the valves: but if any division of a valve be spread out, then much finer *tendons of the third order* come into view, which are given off from the preceding, and are attached to the thin and looser free marginal portions of the division. Their points of attachment to the back of the valve usually lie in straight lines drawn from the tendons of the second order, which give them off, to the very edge of the valve; and their number depends on the breadth of the free marginal portion. Where two divisions of a valve meet at their attached margins a tendon of the first order is always fixed at their point of union, and always gives off tendons of the third order to the adjacent margin of each division; so that when the ventricle contracts the two adjacent divisions must be brought together by the influence of the one set of tendons with which they are both connected. 11. The valves are composed (besides their proper tissue and the endocardium) of the continuations of the tendinous cords which usually spread out like palm-leaves and are interwoven, and of muscular fibres, of which a certain number may be traced, (especially after several days' soaking in cold water), passing from the adjacent wall of the auricle into the interior of each division of the valves, and connecting themselves with the ends of the tendons of the second order in the central portion of the valve. 12. These muscles may be supposed to have the office of keeping the valves tense when, in the contraction of the ventricles, the auriculo-ventricular rings are reduced in size, and the fleshy columns are gradually brought nearer and nearer to each other: but neither they nor the columns can have any share in raising the valves to close the orifice; this must be effected by the blood. When the ventricle contracts the columns contracting with it fix, through the tendons of the first order, the attached margins of the valves; then the blood pressing on their outer surface unfolds them and spreads them before the orifice, into the form of a cone which gradually elongates and becomes narrow, and is flattened as the ventricle empties itself. 13. The purpose of the complicated but regular arrangement of the cords must be to secure the strength of every part of the valve when pressed by the blood. Probably, when unfolded, the dentated edges and the marginal portions are distended and fit into each other. The size also of the valves which appears to be more than enough to close the orifices, reduced as they are in the contracted state of the ventricles, makes it probable that they are not completely unfolded at once in the contraction of the ventricles, but that as the size and position of the ventricle and the size of the orifice change, so different portions are successively unfolded and brought to resist the varying pressure of the blood.

Valentin* has supplied, by laborious investigations, numerous valuable data for the study of the circulation, and has also confirmed most of those already established by Poiseuille and others. Among his deductions are, 1, that in health the quantity of muscle in the right ventricle of man, many mammalia, and birds, is equal to one half of that in the left; 2, that the quantity in the right auricle is about equal to 2-3ds of that in the left; 3, that the muscular power of the right auricle is to that of the left as the square root of the muscular power of the right ventricle to the square root of that of the left; 4, that, in man and the higher mammalia, the absolute force exerted by the left ventricle is equal to 1-50th of the weight of the body; by the right ventricle equal to 1-100th of the same; 5, that the average quantity of blood discharged by the left ventricle is five ounces [an estimate, for which the examinations are insufficient to establish it as certain, but which agrees much better than the old one of two ounces with the time in which the blood makes the round of the circulation; for, according to this estimate, the blood may all pass through the heart in from 41 3-4ths to 62 2-3ds seconds.] 6. That a pressure equal to that of a column of mercury from 1-12th to 1-3d of an inch is sufficient for the effectual closure of any of the

* Lehrbuch der Physiologie, Bd. i, pp. 415-30-36-43, &c.

valves; so that even the weakest action of the heart is, probably, enough for it, provided they be healthy.

To prove that the impulse of the heart depends [but it can be only chiefly dependent] on the contraction of the ventricular fibres, Valentin* cut off the apex of the heart, in several cases, so that the resistance of the blood and the great vessels and the supposed consequent recoil, were prevented. Yet the tilting movement was observed, as much as when the heart was entire. In evidence, also, that the first sound is due to the tension of the auriculo-ventricular valves, he says, that if a portion of a horse's intestine be tied at one end, be moderately filled with water, *without any admixture of air*, and have a syringe containing water fitted to the other end, the first sound of the heart is exactly imitated by forcing more water in. It may be distinctly heard with the stethoscope applied near the tied end of the intestine, at the instant of the water making it tense.

*Arterial circulation.* Some measurements of arteries for the comparison of the respective areas of trunks and their branches, by Dr. E. Hazard, have been published by Dr. Horner.† The mode of measurement and other circumstances are not stated; but the results are, on the whole, confirmatory of those which I obtained,‡ namely, that generally the joint area of branches is rather greater than the area of their trunk.

Dr. Spengler,§ of Etville, has been making experiments on the force of the current of arterial blood, and its variations in the several acts of the heart and respiration, the results of which give strong confirmation of nearly all those obtained by Poiseuille and others. He has used a new apparatus which is, perhaps, less liable to error than the hæmadynamometer, and which can be so adapted to an artery that the amount of lateral pressure exercised by the blood upon its walls may be ascertained without obstructing the current. He has thus obtained the first experimental evidence of what Dr. T. Young and Weber calculated, and the experiments of Poiseuille made almost certain, namely, that the pressure which the current of blood exercises at any part of the arterial system is equal in all directions.

Valentin‖ also has abundantly confirmed the results of the same experiments; and, from numerous micrometric measurements of the diameters of several arteries and of the thickness of their walls, he concludes, that the thicknesses of the walls of any two systemic arteries are in direct proportion to the square roots of the absolute (*hydrodynamic*) forces under which the blood flows in them; ¶ and that the thickness of the wall of the pulmonary artery is to that of the aorta, as the square root of the force of the right ventricle is to that of the force of the left; i. e., as already stated (p. 261) as $\sqrt{1} : \sqrt{2}$.

*Capillary circulation.* Valentin** has estimated the velocity of the capillary circulation in many careful microscopic examinations of frogs' feet during breeding time, and has found the average to be between 1-5th and 1-3d of a Paris line per second; or from ·938 to 1·4 English inch per minute. In the small veins it is about 1-8th faster. [These results agree nearly with those of Hales, who stated the velocity at an inch in a minute and a half: and more nearly still with those of E. H. Weber, who found it 1¼ inch in the minute].

*Venous circulation.* Valentin†† has also, by an apparatus like that used by Poiseuille for estimating the dilatation of the arteries in their pulsation, determined the amounts of the dilatation of the veins near the chest in the act of expiration, and of their contraction in inspiration. In the external jugular vein of the dog the average dilatation is equal to 1-12th of the circumference of the vein:

* Lehrbuch der Physiologie, Bd. i, p. 427, &c.

† Special Anatomy and Histology, vol. ii, p. 172; Philadelphia, 1843. ‡ Med. Gaz. July 8, 1842.

§ Muller's Archiv, 1844, Heft 1. ‖ Lehrbuch, p. 456.

¶ [If this be true, it will follow, since the hydrodynamic forces in different arteries are directly proportionate to the areas of sections of those arteries, or, to the squares of their diameters, that the thicknesses of the walls will be directly as the diameters: but this appears improbable, although the few measurements made may show that the rule is true for the several parts of the aorta and the innominata.]

** Physiologie, i, p. 468.

†† Ib. p. 501.

the enlargement of the portion of vein (an inch and a quarter in length) which was enclosed in the apparatus, was between 1-10th and 1-11th of its previous dimensions.

*Circulation in the lungs.* Mr. Erichsen,* in an essay to prove that the real cause of death after the sudden introduction of air into the veins, is the difficulty of the passage of frothy blood through the pulmonary capillaries, relates this experiment; a pressure equal to that of from one and a half to two inches of mercury is sufficient to drive bullock's blood, deprived of fibrine, through the capillaries of the lungs of a dog recently killed; but if air be previously blown into the pulmonary artery it will require a pressure equal to that of from three to three and a half inches of mercury to force similar blood through the same set of vessels. The pulmonary circulation being thus arrested, the left ventricle receives an insufficient supply of blood, and respiration ceases in consequence of the defective quantity of arterial blood sent to the nervous centres. But, for some time after respiration has ceased and animal life has nearly ceased, the heart continues to act regularly and forcibly; nor do its right cavities ever become so distended as they are in ordinary asphyxia, unless the air have been forcibly blown into the veins.

RESPIRATION.

*Respiratory movements.* In a fourth memoir MM. Beau and Maissiat† have completed their account of the respiratory movements.

That which is chiefly interesting (if not accurate) in this as in their former memoirs, is the account of the actions of the muscles. They, first, rightly represent the oblique and transverse muscles of the abdomen acting together in complex respiration, as the expiratory muscles of the abdomen; like the triangularis sterni and intercostals which they regard as the corresponding expiratory muscles of the chest. In the costo-inferior type of respiration, they act by depressing the ribs; in the costo superior, by drawing in the abdominal walls, and pressing the abdominal viscera against the diaphragm; and in very deep and forcible complex expirations in either type, they act in both these ways. The authors further represent the *recti abdominis* as flexors of the thorax on the pelvis; *not* expiratory muscles; and they add that the fibrous transverse septa serve as bonds for the muscular fibres, which, but for them, might often be separated by the eccentric pressure of the abdominal viscera in expiration. The septa are, besides, supposed to be stretched in deep inferior costal inspirations, and to act by their elastic recoil with other elastic parts in the following expiration; [the recti abdominis may have no part in complex expirations in dogs, in whom the authors examined them, and found them tranquil during cries; for, in dogs, they commonly pass straight from the thorax to the pelvis; but in men, in whom these muscles are usually arched forwards, they may be felt contracting in all forcible complex acts of expiration. By their contractions they probably serve in two ways: 1, by straightening themselves so as to reduce the size of the chest and press up the diaphragm through the medium of the abdominal viscera; and 2, by acting, by means of their transverse intersections, upon their own sheaths which they make tense and fitter to afford fixed points for the action of the oblique and transverse muscles. There can hardly be such an elastic contraction of these bands as is assumed; their tissue is not one which would recoil quickly after being stretched, nor is there any of the inspiratory forces (especially among those admitted by the authors) which would be capable of stretching it, in order to its recoiling]. The *sacro spinalis*, *longissimus dorsi*, and *transversalis colli* are described as extensors of the trunk; not expiratory. The *quatratus lumborum* as the continuation of the infra costales; a lateral flexor of the spine; probably not expiratory.

At the end of their memoir, the authors enumerate the several muscles they have described, according to their functions, as follows: [and I have added signs

* Edinb. Med. and Surg. Journal, Jan. 1844.

† Archives Gén. de Médecine, Nov. 1843. See last Report.

of doubt when their conclusions appear improbable]. *Non-respiratory*—Sacro-spinalis, rectus abdominis (?), serratus posticus superior (?), quadratus lumborum. *Doubtful*—Levatores costarum [inspiratory ?], serratus posticus inferior [expiratory]. *Inspiratory: directly* and *ordinarily*—Diaphragm and scaleni, the one or the others predominating according to the type of the respiration: *direct* and *extraordinary*—sterno-mastoid, pectoralis minor, lower fourth of the pectoralis major, serratus magnus: *indirect*—trapezius and levator anguli scapulæ. *Expiratory: direct and ordinary*—in forcible expiration, intercostals (?), infracostales, triangularis sterni, latissimus dorsi, transverse and oblique abdominal, pyramidalis: *direct and extraordinary*—upper three-fourths of the pectoralis major: *indirect*—trapezius, sphincter ani, levator ani, coccygeus.

*Capacity of respiration.* Many very interesting and practically important results have been obtained by Mr. Hutchinson,* with his *spirometer,* an instrument by which the capacity of respiration is measured by the quantity of air expired in a full and forcible expiration. Among these the chief is the fact of the existence of an intimate relation between this capacity and the height of the individual examined. In 1088 healthy men from five to more than six feet in height, he found the capacities of respiration as follows: in men of 5 feet, 135 cubic inches; of 5 ft. 1 in., 177 c. i.; of 5 ft. 2 in., 173 c. in.; of 5 ft. 3 in., 184 c. i; of 5 ft. 4 in., 193 c. i.; of 5 ft 5 in., 208 c. i.; of 5 ft. 6 in., 204 c. i.; of 5 ft. 7 in., 224 c. i.; of 5 ft. 8 in, 220 c. i.; of 5 ft. 9 in., 229 c. i.; of 5 ft. 10 in., 246 c. i.; of 5 ft. 11 in., 254 c. i.; of 6 ft. 255 c.i.; of upwards of 6 ft. 260 c. i. These numbers are such that it may be generally stated that for every additional inch of height from 5 to 6 feet, eight additional cubic inches of air, at 60°, are given out by a forced expiration. And the results of the examinations are so nearly uniform that disease may be suspected in any man who cannot blow out nearly so many cubic inches as the average of those of the same height, even when, by external measurement, his chest appears to be of full size. Indeed, in general, the size of the chest affords no good indication of the capacity of expiration. The only exceptions among healthy men to the general rule of the direct proportion between the height of the body and the capacity, are in the cases of fat men whose capacity is always low.†

In the ordinary respiration of men, from seventeen to thirty-three years old,‡ Valentin has calculated from the watery vapour contained in the saturated expired air, that the average quantity of air expired in a minute is 400 cubic inches; the extremes, under varying circumstances, being 234 and 686 cubic inches; and the average quantity in one ordinary expiration 31·1 cubic inches, the extremes, in very tranquil and somewhat hurried respiration, being 11·4 and 74 cubic inches. [Mr. Coathupe's estimate of 20 to 25 cubic inches is probably better, for it was drawn from the results of respiration continued during a longer period and with less restraint than those of Valentin's]

*Force of respiratory movements.* From another set of experiments with an instrument something like a hæmadynamometer with a mouth-piece, Valentin§ deduces that the force exerted in tranquil inspiration and expiration is equal to the pressure of a column of mercury from ·13748 to ·3937 of an inch high; and the force of the same acts when violent is equal to the pressure of a column from ·7874 to 1·5748 high.

Mr. Hutchinson‖ finds that the full expiratory force of a healthy man is generally about 1-3rd greater than his inspiratory force. Taking the general rule, among 1200 persons, of various classes, the inspiratory force increases pretty regularly from those of 5 feet high to those of 5 feet 9 in., and then decreases. If the power of the respiratory muscles be a fair measure of the power of the whole muscular system, the men of this latter height might, therefore, be regarded

* Lancet, July 27 and Aug. 3, 1844.

† It was this observation (which has also been made by M. Bourgery, see last Report, p. 11) that thin men have the greatest capacity of respiration, which first led Mr. Hutchinson to the discovery of his law.

‡ Lehrbuch, p. 542.

§ Ib. Bd. i, p. 525.

‖ L. c.

as the strongest. But in four classes of men picked for active service, Mr. H. found the respiratory force greatest in those of 5 feet 7 in.*

*Changes of the air in respiration.* The whole of this subject has been very carefully examined by Valentin and Brunner,† operating on large quantities of quietly respired air: and their results are probably more nearly deserving of implicit confidence than any hitherto published. They are, briefly, as follows: 1. The expired air has always, (even in widely varying external temperatures), a temperature of from 97·25° to 99·5° F.; most frequently the latter temperature. 2. It is always saturated with watery vapour. The quantity of vapour exhaled from the blood in the air-passages may therefore be estimated by subtracting the quantity contained in the atmospheric air inspired, from the quantity which (at the same barometric pressure) would saturate the same atmospheric air at the temperature of 99·5°. And, on the other hand, if the quantity of watery vapour in the expired air be estimated, the quantity of the air itself may from it be accurately determined, being as much as that quantity of watery vapour would saturate at the ascertained temperature and barometric pressure. 3. The chemical changes are due to the simple diffusion of gases taking place between those of the atmosphere and those of the blood. The nitrogen is unchanged. The volumes of oxygen absorbed and of carbonic acid exhaled from the blood are determined by the established laws of the diffusion of gases, so that for 1 volume of carbonic acid exhaled, 1·17421 volume of oxygen are absorbed;‡ or, by weight, for one part of carbonic acid, 0·85163 of oxygen. Now one part by weight of carbonic acid contains 0·72727 of oxygen; therefore, for each part of carbonic acid which is discharged in respiration there is an excess of 0·12436 of oxygen which enters the blood and is disposed of otherwise than in forming the carbonic acid excreted from the lungs; or, by volumes, for each one of carbonic acid, an excess of 0·17421 of oxygen. Hence, if it be known how much carbonic acid a man has exhaled from the lungs in a given time, we may reckon how much oxygen he has in the same time absorbed. Valentin and Brunner have determined that, in a medium of temperature and atmospheric pressure, they each, on an average of six experiments, breathed 562·929 litres of air in the hour, and each in the same time expired 635·8565 grains of carbonic acid containing 173·414 grains of carbon. From this and from their respective diffusion-volumes the hourly consumption of oxygen may be calculated at 541·5 grains; and these results agree very nearly with those obtained by Andral and Gavarret.§ They show that in each hour 69·0575 grains i. e. 541·5—(635·85—173·414) of oxygen are absorbed which are not employed in forming the carbonic acid of the expired air.||

But, notwithstanding these exhalations of carbonic acid, M. Boussingault,¶ from 142 analyses of large quantities of the air of Paris, has confirmed the conclusion already generally received, that the quantity of carbonic acid contained in the air of large towns, is not above the average. The average quantity which he found was 3·97 volumes in 10,000. From the quantity of combustibles consumed,

* The rules established in these valuable papers are especially applicable to the examination of men for military or other active services. They contain numerous other facts of considerable importance in practice, which, however, can hardly find a place in a report of this kind.

† Lehrbuch, Bd. i, p. 547, e. s.

‡ These numbers represent the proportionate diffusion-volumes of the two gases, calculated according to the law of their being inversely as the square roots of their specific gravities. The results of the experiments were so nearly the same that the differences may be safely referred to errors of analysis.

§ See last Report, p. 11.

|| The recent observations of M. Gay Lussac (Annales de Chimie et de Physique, Mai, 1844) directed against the experiments of Magnus, only prove inconsistencies in the results of those experiments. It was always sufficiently evident that the *quantities* of gases supposed by Magnus to exist in the blood could not be right; but nothing has disproved either the fact that such gases do exist therein, or the theory that the carbonic acid is formed in the systemic circulation; and if further evidence were necessary, it is abundantly furnished by the results of Valentin and Brunner's experiments, which show that the proportions of carbonic acid and oxygen that are interchanged are determined, not by their chemical equivalents, but by their diffusion-volumes. It is hardly possible that this should happen unless the carbonic acid were already formed and dissolved in the blood when it arrives at the pulmonary capillaries.

¶ Ann. de Chimie et de Physique, Mars, 1844, t. 85.

in food, fuel, and lighting, in Paris, he estimates the daily produce of carbonic acid at 115,932,871 cubic inches; and the speedy diffusion of such a quantity is not surprising, when it is added that the surface of the ground "within the walls" of Paris measures 1354,229,533 square inches; so that if the whole of the carbonic acid produced in twenty-four hours, were produced in an instant, it would form a layer on the surface less than an inch in thickness.

To this it may be added, on the authority of Mulder,* that the results, similar to the above, which were obtained by De Saussure, as to the quantity of carbonic acid contained in the atmosphere at various times and places, have been confirmed by Verver, whose experiments, like those of Boussingault, were performed by means of Brunner's aspirator. He says also that he has instituted examinations to determine the quantity of ammonia in the atmosphere, and that it is so extremely small, that it is not possible that plants should derive their nitrogen from it; it is not more important, as a constituent of the atmosphere in its relations to the organic kingdom, than many other of the innumerable substances that are exhaled into the air, and brought down again with the rain.†

But if this be true, there must be differences in the quantity of ammonia in the atmosphere of different places, which is very improbable. For Dr. John Davy‡ has found traces of its presence in several samples of rain-water collected at Ambleside; [and in many fogs in England, it may be detected by the action of the air on slightly reddened and moistened litmus paper. In London fogs it is evident to the nose and eyes; and, as in these, so about Ambleside, it is probably brought down with soot, for Dr. Davy describes a layer of carbonaceous matter, like particles of soot, often covering wide extents of the surfaces of the Westmoreland lakes. Such layers are abundant enough on the surface of water in London; after a night of still frost, great quantities of soot might be swept from the surface of the ice. May we not suppose that the minutely-divided carbon which floats in the atmosphere, is constantly disinfecting it by absorbing not ammonia alone, but many other gases, and holding them in its pores till it falls, or is washed down by rain, and yields them to be decomposed by plants?]

It would be beyond the limits of this Report, to enter far on the subject of the purification of the atmosphere from the changes produced by respiration. It must suffice to refer to the papers of Prof. Draper,§ Dr. Gardner,‖ and Mr. Hunt,¶ (especially to that of the first,) on the decomposition of carbonic acid by plants, under the influence of those rays alone of light, which occupy the most luminous part of the spectrum, the orange, *yellow*, and green rays; and to the observations of Wöhler** and Morren,†† on the removal of carbonic acid, and production of oxygen, by certain of the minutest and most abundant infusoria.

### ANIMAL HEAT.

An extensive series of observations has been made by M. Roger ‡‡ on the temperature, of children in health and various diseases.

In nine examinations of infants from one to twenty minutes after birth, the temperature, (observed in these and in all the other cases, in the axilla,) was from 99·95 to 95·45. Immediately after birth the temperature was at the highest; but

* Physiol. Scheikunde, pp. 113, 160.

† Mulder considers that the real source of the nitrogen of plants is in the ammonia formed by the combination of the nitrogen of the moist atmospheric air, contained in the porous earth, with the hydrogen given off from the decaying organic compounds. By a similar process, ammonia is formed by the decomposition of water and atmospheric air in all porous bodies, provided they are moist and exposed to the air at a certain temperature; a similar productinn of ammonia from water and atmospheric air is a part of the process by which nitre is formed in many natural nitre-caves.

‡ Edinburgh New Philos. Journal, July, 1844.

§ Philosophical Magazine, Sept. 1843, and many subsequent parts.

‖ American Journal of Science and Arts, Jan. 1844; and Edinb. New Philosoph. Journal, July, 1844.

¶ Philosoph. Magazine, 1843-4, various parts.

** Annalen der Chemie und Pharmacie, Bd. xlv, p. 206; aud Schmidt's Jahrbuch. Oct. 1843, Bd. xl.

†† In Mulder, Physiol. Scheikunde, p. 117; and Ann. de Chim. et de Phys. Sept. 1844.

‡‡ Arch. Gén. de Médecine, Juillet, Août, &c. 1844.

it quickly fell to near the lowest of those above stated; but, by the next day, it was again completely or nearly what it was before. The rapidity of the pulse and of respiration appeared to have no certain relation to the temperature.

In thirty-three infants of from one to seven days old, the most frequent temperature was 98·6; the average was 98·75; the maximum (in one case only,) 102·2; the minimum, (also observed only once,) 96°·8. All the infants were healthy. The frequency of respiration had no evident or constant relation to the temperature. A few of the infants were of a weakly habit; their average was 97·7, the others were strong, and their average temperature was 99°·534. The age of the infant (in this short period,) had no influence on its temperature; neither had its sex, nor its state of sleep or waking, nor the period after suckling.

In twenty-four children, chiefly boys, from four months to fourteen years old, the most frequent temperature was above 98°·6; the average was 98°·978; the minimum was 98°·15; the maximum 99°·95. The average temperature of those six years old or under, was 98°·798; of those above six years old, 99°·158. The average number of pulsations in the minute was in those under six years old 102; in those above that age 77; yet the temperature of the latter was higher than that of the former, or of younger infants. There was no evident relation between the temperature and the frequency of respiration; nor, in a few examinations, was the temperature affected in a regular way, by active exercise for a short time, or by the stage of digestion.

As already said, in all the examinations from which these results were obtained, the thermometer was held in the axilla; comparative examinations proved that the temperature of the axilla, (though lower than that of internal organs,) was higher than that of any other part of the surface of the skin. Of the other parts examined, the warmest was the abdomen, then in succession, the cavity of the mouth, the bend of the arm, the hands, the feet; of which last, the average temperature, in four examinations was only 87° 35. (These results correspond sufficiently with those obtained by Dr. John Davy.)

In diseased states, (to the illustration of which the greater part of the memoir is devoted,) the temperature of the skin in children may descend to 74°·3, and may ascend to 108°·5. Its range of variation is therefore much greater than in adults, in whom M. Andral found it to vary in different diseases not more than from 95° to 107°·6.

Dr. John Davy* also has contributed some miscellaneous observations on animal heat. They were made with a thermometer placed beneath the tongue. In eight old men and women, all, with one exception, between eighty-seven and ninety-five years of age, the temperature was 98° or 98°·5; therefore not below the average of persons in the like circumstances. But, two observations showed, that in exposure to external cold, the temperature was more reduced than in young persons, in one case to 95°, in the other to 96°·5. A few observations were also made on persons working in rooms at a temperature of 92°; in one case the temperature under the tongue was 100°, in another 100°·5; and in a third, with an external temperature of 73°, it was 99°. The same slight variations of the temperature of superficial parts in accordance with changes of external temperature were shown by repeated observations on a healthy man in the different seasons at Constantinople. By moderate exercise the temperature was raised, (but not above the general average,) on the surface of the extremities, and was not affected in the internal parts.

Some very interesting observations on the effects of hot moist air upon the body were made by M. Constantin James,† at the baths or stoves of Nero, called formerly Posidianæ, near Pozzuoli. The physiological part of his account is, that he had first to traverse a passage, leading to the hot springs, about seven feet high, and three wide, in which the temperature in the upper strata of air was 104°F. in the lower 91° 4. After going along this for about fifty yards, the passage narrowing and winding, the temperature in the different strata became 109°·4, and

* Philosophical Transactions, 1844, p. 57. † Gazette Médicale, 27 Avril, 1844.

98° 6, and was very inconvenient; his pulse increased from 70° to 90 times in the minute. After a few instants, as he went on, his pulse became 120°, his temporal arteries beat forcibly, his respiration was short and panting, his body bedewed with sweat; he was obliged to stop every instant, and put his head to the ground, where he could breathe the least heated air. The temperature had now become 118°·4 in the upper, and 111°·4 in the lower strata, and the atmosphere was filled with dense vapour. Still descending towards the spring, the atmosphere became still more suffocating; his head felt bursting; he was utterly exhausted, and had nearly lost his consciousness; his pulse could not be counted. The temperature of the spring was 185°, while that of the atmosphere was 122°. Summoning all his strength, the experimenter made his way back through the passage, in which he had been for nearly a quarter of an hour, and of which the whole length was about 120 yards. On coming into the cold air he nearly fainted, and staggered like one drunk, till he was relieved by bleeding at the nose; but through the evening his pulse was 100, he was feverish, had ringings of the ears, and a kind of creeping in the limbs. After a good night's rest he was completely recovered. The water from the spring was clear, and not charged with any gas; neither was any deleterious gas mingled with the heated air.

M. James compares these observations with those made on rabbits and other animals, by M. Magendie. The chief results of these experiments were as follows: two rabbits, of the normal temperature of about 102°·2 were placed in two stoves, one at 212°, the other at 140°; the first died sooner than the second, but the temperature of each at the instant of death was the same, namely 111·2°. And the same experiment often repeated, showed that whatever were the ratio at which the heat was applied, the animal died when this increase of nine degrees was attained. In bird's whose normal temperature [in some,] is 111° 2, i.e. the same as that at which mammals die, death ensues upon the same increase of nine degrees of temperature; they die when their blood is at 120°·2.

If when an animal is near dying from the effect of heat, an artery be opened, its blood is as black as that of a vein, it does not coagulate, and does not become bright on exposure. Exposed to dry heat, the animal loses much weight by evaporation before it dies; exposed to moist heat it loses no weight, but dies sooner. In the vapour-baths of Nero, M. James was almost suffocated in a temperature of 112°, while in those of Testaccio, in which the air is dry, he was but little discomforted by a temperature of 176°; and this has been confirmed on many animals. The quantity of weight lost by evaporation in hot dry air is directly proportionate, not to the temperature, but to the duration of the exposure, and the rate of loss is the same during all the times. After death, the lungs and heart are found in the contrary state to that seen after death from cold; they are empty from blood, and it is collected and extravasated at the surface of the body.

### DIGESTION.

*Saliva.* From analyses, conducted on the same plan as those of the blood, (page 254), Enderlin* concludes that the saliva, like the blood, contains no lactate, carbonate, or acetate; but that its alkaline reaction is due to tribasic phosphate of soda, which serves also as the solvent of the mucus- and proteine-compounds. The analysis of the ashes obtained from a very large quantity afforded, in 100 parts:

| | |
|---|---|
| Tribasic phosphate of soda (3 Na. O, P 2, O 5) | 28·122 |
| Chlorides of sodium and potassium | 61·93 |
| Sulphate of soda | 2·315 |
| Phosphate of lime, ,, magnesia, ,, iron | 5·509 |

He believes, from this, that the saliva must take a very important part in digestion.

And it is but reasonable to connect these discoveries of the basic phosphate

* Annalen der Chemie und Pharmacie, Marz 1844.

of the saliva and the acid phosphate of the gastric fluid with the fact observed by Schultz and Lehmann, and more clearly by Dr. Wright, (see last Report, p. 12, and Lancet, 1842-3,) that the alkalinity of the saliva bears a direct proportion to the acidity of the gastric secretion. The observations made this year upon the relations of the phosphate salts in physiology may, probably, be regarded as the most promising of the year's discoveries.

*Palate.* A very excellent and comprehensive dissertation on the nerves of the palate has appeared from Dr. Hein.* The conclusions of numerous experiments (which will be referred to again in the Report on the Nerves), were in many respects similar to those of Volkmann. The soft palate is supplied by four nerves. The mucous membrane of the anterior and upper surface and the subjacent glands receive sensitive filaments from the posterior palatine nerves of the second division of the trigeminus. The lower part of the anterior surface, and the surface of the anterior arch, are supplied by gustatory branches of the glosso-pharyngeal, which also gives gustatory branches to the middle part of the lower region. The whole posterior surface and posterior arch receive sensitive fibres from the pneumogastric and accessory. The second division of the trigeminus also sends filaments (by the middle palatine nerve) to the levator and azygos muscles; but these are probably centripetal nerves. Of the muscles, the tensor and azygos receive (besides these centripetal fibres) motor fibres from the pneumogastric and accessory; the tensor receives fibres of both kinds from the otic ganglion, and, probably, the internal pterygoid branch of the third division of the trigeminus: the palato-glossus is similarly supplied with both kinds of fibres by the glosso-pharyngeal, and the palato-pharyngeus by the pneumogastric and accessory. The stylo-pharyngeus is supplied by the glosso-pharyngeal.

*Œsophagus.* Two new muscles connected with the œsophagus are described by Professor Hyrtl† of Prague, [and I can bear witness to at least the general truth of his description ] One serving, perhaps, to diminish the movement of the left bronchus when the food passes it,) is connected by a broad base with the posterior wall of the bronchus. and thence extends to the left wall of the œsophagus, and mingles with its longitudinal fibres, continuing two or three inches with them. The other (for fixing the œsophagus below the point where it crosses the bronchus,) takes its origin from the left wall of the posterior mediastinum behind the aorta, over which it turns to reach the œsophagus. The first muscle is named by him broncho-œsophageal, the second pleuro-œsophageal.

### STOMACH.

*Gastric digestion.* A very extended examination of the phenomena of gastric digestion has been made by M. Blondlot.‡ The chief subject of experiment was a dog, in which he maintained, without affecting the health, a fistulous opening into the stomach for more than two years. His examinations have furnished many new and important facts, and have confirmed those of Dr. Beaumont made on Alexis St. Martin in nearly every point.

*Secretion of the gastric fluid.* Like Dr. Beaumont, he has found that no mechanical irritation of the interior of the stomach will produce a secretion of nearly so much or so pure gastric fluid as the introduction of food. By mechanical irritation he could never obtain more than 180 grains of fluid, and this was mixed with mucus: when food was introduced, the gastric mucous membrane immediately became turgid, and yielded ten times as much digestive fluid pure. In this turgid state, also, both these observers agree that the mechanical irritation, which was ineffectual under other circumstances, greatly increased the secretion of the gastric fluid. In the turgid state, moreover, chemical irritation, such as was pro-

* Müller's Archiv, Heft iii, iv, an inaugural prize essay; Heidelberg.

† Wigner Zeitschr. i, ii, 1844; and Schmidt's Jahrbucher, Sept. 1844.

‡ Traité Analytique de la Digestion, 8vo; Paris, 1844. Of course so novel a mode of experimenting has been imitated. M. Payen and Dr. Basson (Froriep's N. Notizen, Feb. 1844) have published the results of their experiments; but there is nothing in them which has not been long known.

duced by putting pepper, salt, sugar, &c. on the food, produced a still greater effect than mechanical irritation did; and so did alkalies, but acids seemed to have a contrary influence. The act of digestion and of secreting gastric fluid was not, in either set of observations, found to be attended by an increase of the temperature of the stomach. The statement of Dr. Beaumont is also confirmed, that, cæteris paribus, the quantity of gastric fluid secreted is directly proportionate to the quantity of food taken, provided that quantity, however great, is not more than the organism requires ; and the quantity secreted appears to bear a close relation to the degree in which the food taken is digestible or the contrary.

Its quantity is also apparently influenced by impressions made on the mouth: e. g sugar introduced into the dog's stomach, either alone or mixed with human saliva, excited a very small secretion ; but when the dog had himself masticated and swallowed it, the secretion was abundant.

Some strange experiments on the secretion of the stomach are also related by M. Claude Bernard.* The results of the strangest are these :† 1. The mucous membrane of the stomach, during fasting, is not alkaline nor neutral: if the mucus be wiped off it, it gives an acid reaction. 2. Its acidity, even during digestion, is quite superficial; if a part of its surface be scraped, the acidity of that part disappears. 3. It is acid, even in the young fœtus ‡ 4. If arterial blood, just drawn from one dog, be injected into the coronary artery of the stomach of another, just killed, and having the stomach laid open, there will gradually ooze from the gastric mucous membrane a transparent dew-like fluid, with an acid reaction. If a small quantity of ferrocyanuret of potassium have been added to the blood, it also will be detected in the oozing fluid. 5. A dog was well fed ; half an ounce of weak solution of ferrocyanuret of potassium was injected into its jugular vein, and half an hour after, (having fed again in the interval,) it was killed. The partially-digested food in the stomach, and the internal surface of the stomach, struck a blue colour on the contact of a solution of salt of iron ; but, with the exception of the urine, no one other fluid, whether secreted or exhaled, and no tissue of the body afforded a similar indication of the presence of any of the injected salt. (?) 6. If the same salt be injected into the blood of a fasting animal, it is not effused into the stomach. 7. If ferrocyanuret of potassium be injected into one jugular vein of a dog, and protosulphuret of iron into the other, they do not appear to unite in the blood, nor in any tissue or organ [or fluid ?] of the body, except the gastric fluid : the partially-digested food in the stomach is the only thing that is coloured blue. (?) 8. If lactic, phosphoric, butyric, or acetic acid be injected into the blood, it is found in the stomach. 9. If alkaline solutions of magnesia and iron be so injected, those bases are never found in the gastric fluid. 10. If salts—such as lactate of iron, or butyrate of iron or magnesia—be injected, the acids are found in the gastric fluid, and the bases pass into the urine. If cyanuret of mercury be injected, hydrocyanic acid is smelt in the stomach, but the mercury is never found in it. 11. If a mineral salt which cannot be decomposed in the blood be injected, it passes entire into the stomach : e. g. the ferrocyanuret of potassium and protosulphate of iron.

*Analysis and properties of the gastric fluid.* The most remarkable results of M. Blondlot's investigations relate to the composition of the gastric fluid, and different as his conclusions may be from those usually received, yet the large quantity of fluid he was enabled to collect in a purer state than any one hitherto has collected it, entitles his account to every consideration. He very

* Gazette Médicale, Mars 16, 1844.

† Part only are quoted, and many of these need confirmation. None of the author's deductions are mentioned; for these are adapted to the opinion that the gastric mucous membrane has no glands, except such crypts as are found in the intestines, and that it has villi identical in structure with those of the intestine. The experiments which are here omitted are explicable by physiological facts, familiarly known in most parts of Europe, but which the author, assistant to M. Magendie, appears not to have heard of.

‡ M. Bernard says he found it acid in a human fetus of about seven weeks ; but the separation of the stomach from the rest of the digestive canal does not begin till after two months. Perhaps, he means seven months.

carefully distilled on a sand-bath 3875 grains of pure gastric fluid obtained after feeding his dog with raw meat; he repeated the distillation, and repeated the whole experiment, several times, with the gastric fluid of other animals as well as of the same dog, and the constant result was, that the product of the distillation did not once exhibit the slightest acid reaction; but the residue in the retort was always strongly acid. It was thus proved that the acid of the gastric fluid cannot be either the hydrochloric or the acetic, for both these are volatile at the boiling point of water, and would have distilled over.

A further proof that it is neither of these nor lactic acid, was furnished by the fact that no effervescence is produced when chalk, marble, or any other carbonate of lime is added to the gastric fluid; and it was this fact which chiefly led M. Blondlot to his conclusion, that the true and almost only source of the acidity of healthy gastric fluid is the presence of superphosphate and biphosphate of lime. The evidence which he gives in addition to the above is: 1st, there is no acid salt, except this super-phosphate of lime which could retain its acidity and remain in contact with carbonate of lime without exciting decomposition; 2d, sulphuric acid, added to gastric fluid, produces an abundant precipitate of sulphate of lime, and oxalic acid a similar one of oxalate of lime. 3. Potash, soda, ammonia, and lime-water, produce abundant precipitates of neutral phosphate of lime. 4. The calcined ash of gastric fluid was not deliquescent, was dissolved without effervescence by a few drops of hydrochloric acid, with which it formed chloride of calcium; it had, therefore, contained neutral phosphate of lime, the excess of the acid having been decomposed in the calcination.

The general conclusion of his analysis is, that the gastric fluid is composed of ninety-nine parts of water, with one part of super-phosphate of lime, super-phosphate of ammonia, chloride of sodium, mucus, an aromatic, and a peculiar, principle. Similar results were obtained from the analysis of the gastric fluid of several animals.

For further evidence that the acid reaction of the gastric fluid depends on these acid phosphate salts alone, M. Blondlot has completely examined the question whether, during healthy digestion, lactic acid is ever formed by transformation of the food in the stomach. His conclusion is that neither it, nor a transformation of sugar into starch, nor any kind of fermentation takes place. He has often analysed the fluid expressed from food which had remained for various lengths of time in the stomach, and never found the least trace of lactic acid; and the reason he assigns for its absence is, that the acid of the gastric fluid prevents it, just as other acids prevent the lactic fermentation from taking place in a solution of sugar, provided they are present in proportion sufficient to give the solution a degree of acidity equal to that which it would acquire if the lactic acid were formed in it. In confirmation of this he shows, by numerous experiments on ruminants and birds, that lactic acid is formed by the transformation of the sugar of their food in all those parts of the digestive canal in which the food is delayed without the presence of an acid; namely, in the first and second stomach of ruminants, the crops of birds, and the cœcum of man and other animals. He first proves that the acidity often observed in the food taken from these cavities is not due to any secretion from their walls. He fed, for four days each, sixteen sheep and goats, and several pigeons and chickens with different kinds of food containing no sugar; and in every instance the portions of food which were found after twelve hours fasting, in the first stomach, or the crop, were not acid, but alkaline, proving that the walls of these cavities secrete an alkaline fluid. On the other hand, when, the other circumstances being the same, as many ruminants and fowls were fed on food containing sugar, the portions of food found in the same cavities were always acid, and, in the case in which they were analysed, the acid obtained was the lactic.

In regard to the cæcum he states that its contents are never more acid than those of the small intestines, except when the animals examined have had sugar in their food; from which, and the absence of any proof that the cœcum secretes an acid fluid, he believes that the acidity often found is due to a portion of the sugar of the food which has not been absorbed, and has undergone the lactic acid fermentation in the cæcum.*

These experiments are confirmed also by those of Mr. Ross,† who finds that rabbits fed on farinaceous food have lactic acid in their small intestines, though it is not found in their stomachs. They appear to be contradicted by those of Tiedemann and Gmelin, who found acid in the crop of a pigeon which had fed for several days on nothing but meat: but M. Blondlot shows that this acid had probably flowed from the stomach into the crop after death; an accident which happened in his experiments when means were not used to prevent it.

Besides these experiments concerning the chemical properties of the gastric fluid, M. Blondlot relates others, which add to the evidence already known, that the real digestive property of the fluid depends, not on its obvious chemical qualities, but on an organic principle. If exposed to a temperature between 104° and 122° F. or higher, it entirely and irrecoverably loses its digestive powers, although apparently, and as to analysis, unchanged. Kept from the air, the gastric fluid retains its active properties for at least two years; but, exposed to the air and a moderate temperature, it putrefies in five or six days, although the chyme which it forms from nitrogenous organic substances may be preserved for two or three months without apparent change. The precipitation of all the lime which it contains does not affect its activity; neither are its chlorides indispensable; but whatever much alters its organic constituents, (such as heat, strong alcohol, or strong acids,) or removes them, (such as animal charcoal, tannic acid, chlorine, or acetate of lead,) destroys all the digestive properties.

*Digestive properties of the gastric fluid.* Some singular evidence of these is furnished by MM. Bernard and Barreswill‡ who have found that nutritive substances injected in simple aqueous solution into the blood are not assimilated: but are assimilated if dissolved by the aid of the gastric fluid. Among other experiments are these: portions of cane sugar, albumen, and gelatine, seven and a half grains of each, and severally dissolved in water, were injected into the jugular veins of three dogs. Three hours after, the urine of each was examined, and in each the injected substance was found. Under other similar conditions, the same quantities of the same substances dissolved in gastric fluid were injected, and three hours after, gelatine was detected in the urine of the dog into whom it had been injected, but not a trace of albumen or sugar in the urine of either of the others. Three dogs were then fed exclusively and respectively on gelatine, albumen, and sugar; and the first alone could ever be detected in the urine. The authors fed themselves in the same way and obtained the same result; and they conclude (as others presently to be mentioned do) that gelatine is not assimilable and therefore not nutritive.

Among the experiments which M. Blondlot§ made to determine the mode in which the gastric fluid, in or out of the stomach, acts on different animal substances some afforded novel and interesting results: A. He shows that coagulated albumen owes its long resistance to the digestive fluid only to its compact form. When coagulated in very fine particles (as by pouring white of egg beaten into a froth, into boiling water) it is digested as quickly as soft fibrine. B. He adds further evidence that the action of the stomach in coagulating milk is not due peculiarly to its digestive principle, but to its acid, which acts like the lactic acid developed from the sugar under the influence of rennet or any other decomposing azotized compound. C. The effect of the gastric fluid on bones, observed both on the bones in their entire state, and on their animal and inorganic constituents separately, is, that, first, it very slowly disintegrates the animal matter, attacking them from the surface, and then, also very slowly, disintegrates and reduces the earthy matter into a fine chalky powder, but without either dissolving or decom-

Traité Analytique de la Digestion, pp. 91-104. † Lancet. Jan. 20 and Feb. 10, 1844.
Gazette Médicale, 27 Avril, 1844; Report from the Acad. des Sciences, seance du 22 Avril, 1844.
Loc. cit. pp. 254-383, &c.

posing it. The earthy matter not being dissolved proves that no hydrochloric acid had acted upon it: and in its minutely divided state it all passes through the intestines and is discharged with the fæces.

The results of many more of his experiments of this kind are interesting. They confirm Mr. Beaumont's, and appeared to M. Blondlot to show that, of all the simple alimentary substances, those which are fluid at the ordinary temperature of the stomach, and those which are easily soluble (in the ordinary manner of solution) in its secretion, such as fluid albumen, sugar, gum, pectine, &c., are at once absorbed by the veins; and that others, which are not liquid nor easily soluble, such as fibrine, coagulated vegetable and animal albumen, caseine, gelatine, &c., are, in only a very small proportion, if at all, dissolved, the action of the gastric fluid on them being limited to the softening of them, so that they are reduced into very minute particles which (out of the stomach) appear like a very fine precipitate. The same general rule is said to be observed in the digestion of the compound alimentary substances, both animal and vegetable; the fluid and easily soluble parts cannot be said to be digested, for they are at once absorbed by the stomach; the rest are softened and reduced into very minute particles, which are carried into the intestines, without any change in their chemical constitution, and are, in this state, absorbed by open mouths of the lacteals, visible with the naked eye, at the extremities of the villi. (!) This act of softening is, in some cases, due merely to the acid of the gastric fluid; e. g. in the case of parenchymatous tissues, and succulent fruits and roots, which are similarly softened, at the same temperature, in acidulated water; in the cases of fibrine, coagulated albumen, &c., it is the effect of the *peculiar* mode of action of the gastric fluid.

In any case, chymification is, in M. Blondlot's opinion, no *solution*, but a *division* of the aliment; it undergoes no kind of decomposition.

*Influence of the pneumogastric nerves upon digestion.* M. Bernard* has instituted fresh experiments to determine this still-debated question, making use of the artificial fistulous openings into the stomach, invented by M. Blondlot. A dog's digestion had been thus watched for eight days, and had always been well effected. On the ninth day, after a day's fast, M. Bernard sponged out the stomach, which contracted on the contact of the sponge, and at once secreted a large quantity of gastric fluid; he then divided the pneumogastric nerves in the middle of the neck, and immediately the mucous membrane, which had been turgid, became pale, as if exsanguine, its movements ceased, the secretion of gastric fluid was instantaneously put a stop to, and a quantity of ropy neutral mucus was soon produced in its place. After this, no digestion was duly performed, and milk was no longer coagulated; raw meat remained unchanged, and the food (meat, milk, bread, and sugar, which the dog had before thoroughly digested) remained for a long time neutral, and at last acquired acidity only from its own transformation into lactic acid. In the stomachs of other dogs after the division of the nerves he traced the transformation of cane-sugar into grape-sugar in three or four hours; and in ten or twelve hours the transformation into lactic acid was complete. In others, when the food was not capable of an acid transformation, it remained neutral to the last. In no case did any part of the food pass through the peculiar changes of chymification. In a last experiment, he gave to each of two dogs, in one of which he had cut the nerves, a dose of emulsine and half an hour after, a dose of amygdaline (substances which are innocent alone, but when mixed produce hydrocyanic acid). The dog, whose nerves were cut, died in a quarter of an hour, the substances being absorbed unaltered and mixing in the blood: in the other, the emulsine was changed by the action of the gastric fluid before the amygdaline was administered, and it survived.

*Act of vomiting.* A case is related by M. Lepine† of Châlons sur Saône, proving the partial influence of the stomach in vomiting. The patient's abdomen

* Gazette Médicale, Juin 1, 1844; from the Report of the Acad. des Sciences, seance du 27 Mai, 1844.

† Bulletin de l'Académie de Médecine.

was torn open by a horn, and the stomach was wholly protruded. For half an hour it was seen repeatedly and forcibly contracting itself, till by its own efforts it expelled all its contents except the gases.

Mr. Anderson,* to prove that the diaphragm is not, as Dr. Marshall Hall supposes, inactive in vomiting, gave tartar emetic to two dogs, and, when sickness commenced, he opened the trachea, and at the same time introduced his finger into the abdomen so as to feel the state of the diaphragm. During each effort of vomiting, the diaphragm became tense and rigid, and descended towards the abdomen.

[But neither these nor any other experiments prove that the diaphragm actively compresses the stomach in vomiting, by descending towards the abdominal cavity. Indeed no experiments in which the trachea is opened, can illustrate the action of the diaphragm when, as in vomiting, the glottis is closed; for, in the former case, the diaphragm is free to move either way; in the latter, it cannot move at all without either expanding or compressing the air in the lungs. The true explanation of the act of vomiting must, I think, be intermediate between that which supposes the diaphragm to be one of the muscles actively compressing the stomach, and that which supposes it to be inert. The inspiration which usually precedes the act of vomiting is terminated by the closure of the glottis; after this the diaphragm cannot descend further, except by expanding the air in the lungs, but it remains fixed in its position, and serves as an unyielding surface against which the stomach may be pressed by the contracting abdominal muscles. This position of the diaphragm might be nearly maintained, though it were relaxed, for, if the glottis remained shut, the diaphragm could not be raised except by compressing the air in the lungs. But Mr. Anderson's experiments, as well as several other facts, make it more probable that the diaphragm continues in the act of contraction; or rather, since in his experiments it descended when the trachea is open, we may conclude that when the trachea and glottis are shut, it would not descend, but would remain, as other muscles often do, in the rigid and resisting state of contraction, so as to afford a completely fixed and firm surface for the stomach to be compressed against. And this continuance of the contracted state is proved by rupture of the diaphragm in vomiting, for this rupture is not from over extension of the whole muscle, but from some of the fibres or parts of fibres contracting so vigorously as to tear others which are contracting less or not at all.

A condition essential to vomiting, but not sufficiently considered, is the relaxation of those oblique fibres of the stomach, which like a sphincter embrace the cardia. Unless it be relaxed, no vomiting can take place; for when contracted, they can as well resist all the force of the contracting expiratory muscles as the muscles of the glottis can resist it in the act of straining. The activity of the stomach in M. Lepine's case shows that its movements may be associated with those of the abdominal muscles; and, probably just as coughing, sneezing, &c., are perfect when the relaxation of the muscles which closed the glottis is exactly coincident with the contractions of the expiratory muscles, so is vomiting perfect when in exact coincidence with the same contractions the oblique fibres of the cardia are relaxed.]

### LIVER.

*Structure.* Some valuable papers on this subject have appeared from Müller,† E. H. Weber,† and Dr. Kronenberg,† the general effect of which, (though those of the last two are written with an opposite intention,) is to confirm in a remarkable degree the description by Mr. Kiernan. The amounts given by Weber and Kronenberg, though written independently, are in almost everything alike. Especially, they agree with Mr. Kiernan in describing the hepatic ducts as commencing in very fine networks, which, they add, are interlaced with the capillary networks between the portal and hepatic veins. They demonstrate these networks in both the uninjected and the injected state. E. H. Weber's

* London and Edinb. Monthly Journal of Medical Science, Jan., Feb., March, &c. 1844.
† Müller's Archiv, 1844, Heft iii.

account is that the blood capillaries are from 1-1463th to 1-1959th of an inch in diameter, forming a solid uniform network, with meshes not wider than the vessels themselves; and that the distance through which blood has to pass from the smallest portal to the smallest hepatic veins is from about 1-70th to 1-80th of an inch. The meshes of the blood-capillary network are occupied by the interlacing network of hepatic ducts. These are smaller than any other gland-ducts yet known, being from 1-900th to 1-1340th of an inch in diameter, and have no capillaries on their walls. Their network extends uniformly, and without any division according to lobules, through the whole substance of the liver, and its meshes are very small. There is no anastomosis between the blood-vessels and the ducts; but they are in contact on every side, each filling up the meshes of the network formed by the others, and both together filling every space, and forming the whole substance of the liver, except when large vessels, nerves, &c. run into it.

Full accounts are given of the modes in which the demonstration of these things are obtained. Among them are injections (of necessity only very partial) of the bile-ducts; and these demonstrate, according to Weber, another form of bile-ducts, which are found imperfectly developed on the surface of the transverse and longitudinal fissures, the edges of the gall-bladder, and especially (as Mr. Kiernan also showed) at the connexion of the left lobe and the left lateral ligament. In these parts are networks of comparatively large branches of ducts, beset by cells, and having many branched appendages, which terminate in closed ends filled by cells, and which Weber names *vasa aberrantia* of the liver.

[It does not appear that Weber and Kronenberg have made more complete injections of the hepatic-duct plexuses than Mr. Kiernan did, whose demonstration of this arrangement, so far as injections are concerned in it, is as satisfactory as theirs; for all confess the injections to have been very partial. The chief new evidence for this mode of arrangement is afforded by the microscopic examination of the uninjected ducts. I had a fortunate opportunity for confirming, to some extent, the account already given, in examining parts of a liver last summer, from a case of intense jaundice. The case was of a kind not very unfrequent, in which jaundiced persons die with coma or delirium, and other rapidly supervening signs of cerebral disturbance, and in which, after death, the liver is found pale, or orange-coloured, small, soft, but tough, generally or in most parts nearly bloodless, and with the minutest bile-ducts, in some parts, gorged with bile, although the large ones are not closed nor apparently obstructed; so that sometimes parts of the liver stand out from the rest, of a deep orange or olive colour. In this case, the distended ducts were easily traceable in thin sections of the liver, with a single lens of 1-10th inch focus; and they appeared tortuous, and freely anastomosing, so as to form an irregular network with very small meshes. They appeared filled, not with fluid bile, but with bile-cells; and these, as seen with a higher power, were all pale yellow, and spotted here and there with brilliant yellow points and granules; in some also the nuclei appeared peculiarly bright yellow.]

The chief point in which these accounts differ from Mr. Kiernan's is in denying that the component parts of the liver are arranged in lobules. This has also been denied by Henle and Mr. Bowman, who agree with Weber and Kronenberg in describing the capillary networks as *solid,* (i. e. extending uniformly through the liver.) They also all deny the existence of any fibro-cellular partitions dividing the liver into lobules, and even the existence of more fibro-cellular tissue than serves to invest the larger vessels, &c. of the liver. They deny also that there are any such *interlobular* veins and fissures as Mr. Kiernan described, and state that the smaller branches of these veins communicate by branches only just larger, if at all larger, than capillaries.

Müller's paper is written chiefly for the purpose of maintaining the old view of the lobular arrangement of the liver, and contains many facts which had long appeared to me to afford satisfactory evidence of its truth. He justly observes that the complete injections of the blood-capillaries, on which the objections to the lobular arrangement of the larger vessels are founded, are not the best preparations for demonstrating the distribution of the larger vessels, since these are sure to be concealed by the full capillaries. In less complete injections, they may be traced, as Mr. Kiernan describes them, though not usually with that stiff uniformity in which, for clearness' sake, they are represented in his diagrams. But, without injections, the lobular divisions of the liver may be seen, especially in the pig's liver, in which, as Müller exactly describes it, the whole natural surface, as well as the surface of every secretion, is marked by white lines inclosing angular spaces, which lines are no arteries (as they are supposed by Kronenberg), but the ends of membranous septa of cellular tissue, which form distinct capsules round each lobule, and, altogether, divide the whole liver into minute spaces, so that when the glandular substance inclosed within these capsules is scraped away, they remain like a fine honeycomb, composed of oval cells, about a line in length, and half a line wide. [The general truth of this description can be easily seen in the pig's liver, and traces of the same arrangement in the human liver. The only point in which I think Müller is wrong is in describing the partitions as formed of fibro-cellular tissue. If one be cut from the interior of the liver, it will be found covered on both sides with hepatic cells and granules, which adhere to it much more firmly than those in the interior of the lobule do to one another. When these are scraped off, there remains a very thin and tough membrane, in which there are only a few filaments of fibro-cellular tissue, and which appears to be composed of a very dense network or networks of vessels, with gland-cells still adhering among them. The appearances presented in the pig's liver are such as to indicate that its lobules are by no means generally or uniformly traversed by plexuses of ducts; in their interior they appear to contain only large nucleated biliary cells, with various granules loosely arranged: the ducts appear only in the walls of the lobules.]

Müller adds to these evidences, that, if portions of liver be macerated for eight days in vinegar, the lobules may be easily separated from each other, and all will present smooth surfaces; and that, though the lobular structure seen in most vertebrata is absent in some fish, yet in several of the plagiostomatous fish it is shown by the arrangement of black pigment-cells, which everywhere follow the arrangement of the interlobular substance, so that the surface and sections of the liver exhibit islands of yellow substance, inclosed by dark lines.

*Secretion and properties of bile.* A series of experiments by Schwann* has led to the distinct conclusion of the bile being indispensable to life. They consisted in removing a portion of the common bile-duct, and establishing an external fistulous opening into the gall-bladder, so that the bile might be naturally secreted, but be discharged externally, and not permitted to enter the intestine. Their general result was, that, of eighteen dogs thus operated on, ten died of the immediate consequences of the operation (by peritonitis and other affections aggravated, probably, by the want of bile): and, of the remaining eight, two recovered and six died. In the six which died, death was the result of nothing but the removal of the bile; after the third day, they daily lost weight, and had all the signs of inanition, e. g. emaciation, muscular debility, uncertain gait, falling of the hair. They lived from seven to sixty-four days† after the operation; and the inanition was the greater the longer they survived. Young dogs appeared to die rather sooner than old ones. Licking the bile as it flowed from the fistula and swallowing it, had no influence on the consequences of the operation. In the two dogs that recovered, the importance of the bile was equally well shown; for in these it was found, when they were killed, that the passage for the bile into the intestine had been restored; and the period of its restoration was distinctly marked by their weight (which had previously been regularly decreasing) being augmented and continuing to increase till it amounted to what it was before the operation; and also by the fistulous opening into the gall-bladder healing and the discharge of bile ceasing.

Schwann says he is engaged in further and minute examinations to prove in what way the bile serves its important purpose; and these will probably prove how far several theories respecting it (of which not a few have appeared this year) are true or false.

The chemical composition of the bile has been the subject of careful examina-

* Müller's Archiv, Heft ii, 1844.

† One lived two months and a half; but it is not impossible that the bile-duct was for a time restored.

tion in the Giessen laboratory by Drs. Theyer and Schlosser.* They obtained the bile in what they regard as the perfectly pure state, by evaporating that of the ox, immediately after death, to the thickness of an extract; dissolving it in common alcohol and adding alcohol till all the mucus was separated; treating the clear solution with animal charcoal till all the colouring matter was removed; then distilling off the alcohol and washing the residue repeatedly with ether, till no more fatty matter could be separated from it: and, lastly, evaporating to dryness. Elementary analyses were made of this pure bile, and of its combination with oxyde of lead. The latter was formed by mixing an aqueous solution of pure bile with a diluted solution of acetate of lead; it formed a white ropy plaster-like substance, in which it was proved that the organic substance of the bile remained complete and undecomposed, by reproducing it in its combination with soda. The compound thus formed by separating the organic principles from the combination with lead and uniting it with soda, was in no material respect different from the pure natural bile; so that it was quite evident that the substance which is united with the soda in the bile is (as Demarçay and Dr. Kemp already maintained) a peculiar organic acid. To separate this, in a pure state, various means were used, but the only satisfactory plan was by decomposing the salt which it forms (as already said) with oxyde of lead, by passing (with several necessary precautions) sulphuretted hydrogen through an alcoholic solution of it, and filtering and evaporating the remaining solution. The elementary analysis of the acid then obtained in the separate state agreed with those made of it in its combinations (both natural and artificial) with soda, and with oxyde of lead.

The bilic acid (Gallensäure) thus separated, agrees completely with the *bilifellic acid* of Berzelius; (his *bilin,* the authors regard as pure bile, or bile with an excess of bilic acid); it corresponds also to Demarçay's *choleic acid;* and Kemp's *bilic acid*† is the same, not completely separated from its combination with soda. Thenard's *picromel* and Gmelin's *sugar of bile,* and Berzelius' *bilifellinic* acid, are also this same *bilic acid,* more or less imperfectly separated, and the authors adduce the identity of composition in all the samples of bile that are examined as a proof that it is not, like the urine, a fluid by which a variety of morbid and accidental substances are separated from the blood—a compound of various and uncertain materials,—but a fluid separated by a true process of secretion under the determinate and regular influence of its secernent gland.‡

*Pancreas.* M. Blondlot§ says that having obtained three or four grammes of pancreatic fluid from the duct of a large dog, and examined it by means of an electric current, he could find no trace of albumen in it. He considers it to be of the same nature as the saliva, which he holds to be only a common mucous fluid, a kind of detritus or *caput mortuum* serving no active part in digestion, but merely protecting the organs on which it lies (!)

*Fæces.* The analysis of the ashes of firm human fæces by Enderlin|| yielded, in 100 parts:

| | |
|---|---|
| Chloride of sodium and alkaline sulphate | 1·367 |
| Tribasic phosphate of soda | 2·633 |
| Phosphate of lime and phosphate of magnesia | 80·372 |
| Phosphate of iron | 2·091 |
| Sulphate of lime | 4·53 |
| *Kieselerde* | 7·94 |

From the absence of carbonate of lime he deduces that the fæces could contain no choleic acid.

* Annalen der Chemie und Pharmacie, Oct. 1843. Translated in the Medical Times, Feb. 3, 10, 1844. Additions in the Annalen of May, 1844. † See last Report, p. 13.

‡ For an account of the mode in which Dr. Platner has prepared crystallized bilic acid and neutral bilate of soda from ox-bile, see Müller's Archiv, 1844, Heft ii, and Poggendorf's Annalen, Juli 1844. On the Analysis of the Bile of the Astacus fluviatilis, and some other Crustacea, see J. F. G. Schlemm 'De hepate ac bili crustaceorum,' &c.; Berolini, 4to. The bile of the Astacus is acid, and contains no bilin. In the same dissertation there are general confirmations of the received doctrine of the development of secernent cells, and a minute account of the nerves of the liver in the astacus and helix pomatia. See also some account of Mr. Ross's observations at p. 272.

§ Loc. cit. p. 124, &c. || Annalen der Chemie und Pharmacie, Mars 1844.

### ABSORPTION.

*Lymphatic and lacteal absorption.* A systematic work on the lymphatics has been published by Dr. Herbst.* He considers (as M. Bouisson also does, and as Tiedemann and others may be said to have considered) that a process of secretion is combined with that of absorption in the extremities of the vessels. [But the opinion is maintained on very imperfect evidence; for neither of its authors is acquainted with the best accounts of the structure of the villi, or with the physiology of secretion, as an act performed by cells. Some other singular opinions are maintained in this work; but I give only those new results in matters of fact stated to have been obtained from experiments; and even of these, it is necessary to say, that the evidence, especially that derived from the microscope, is not altogether satisfactory.†]

1. The coagulability of the lymph is directly proportionate to that of the blood; and is probably due to coagulable matter passing from the latter into the former. 2. Blood-corpuscles are a common constituent of lymph; and their number is greatly and proportionately increased in all cases of unusually active circulation, congestion, or inflammation, whether local or general. In the former case, they pass in abundance into the lymphatics of the congested part. 3. When fluids are injected into the blood-vessels in quantity sufficient to distend them, the injected substance, (whether blood, milk, water, gelatine, starch, or whatever it may be) may be almost directly afterwards found in the lymphatics. And this same result is obtained, whether the injection be made during life or soon after death; nor is it only the fluid part of that which is injected which passes into the lymphatics; the solid parts also, such as the blood- and milk-corpuscles and the starch-granules, pass unchanged (though in less proportion) into both the lacteals and the lymphatics. Nineteen experiments are related in proof of these statements: the author ascribes the result to a transudation different only in degree from that which is normal. 4. More than twenty experiments are detailed at great length to prove (chiefly by microscopic evidence) that the lymph-corpuscles found of various sizes (from $\frac{1}{10}$ to $1\frac{1}{2}$ of the size of a blood-corpuscle) in the thoracic duct, are not essentially different from those in the true lymphatics and the mesenteric lacteals, nor from those of milk (!) and of chyme formed from fatty substances: (!) and that therefore the various corpuscles of chyme and milk may be considered to be absorbed entire and unaltered by the lacteals of the villi, and to be thence transmitted to the blood, in which they may also be found unaltered. 5. Another large series of experiments is related to prove that colouring matters (chiefly indigo), salts of potash, lead, &c. and starch in imperfect granules, are rapidly absorbed by the lacteals and by the lymphatics of the stomach. [But there was nothing in the mode of performing them by which it can be explained why their result was different from that obtained by others, who, in similar experiments, have found no absorption of the same substances: they therefore need only be referred to.] 6. Many of the experiments in which the preceding conclusions are founded, and some others purposely made, give evidence (as the very first observations by Asellius‡ do), that, for some time after apparent death, the lymph and chyle continue to be moved onwards by the peristaltic movement of the digestive canal, and by the contraction of the walls of their own vessels; and that also for some time after death, absorption itself is carried on, for considerable quantities of fluids injected into the stomachs of recently-killed animals which had fasted a long time, were carried into the thoracic duct.

* Das Lymphgefässsystem und seine Verrichtung; Göttingen, 1844. 8vo.

† For example, it is often stated that after lymphatic vessels had lain in water till all the colour was taken out of their coats, *unaltered* blood-corpuscles were found in their contents: these are also so described in lymph diluted with water.

‡ De Lactibus seu Lacteis' Venis.

A few facts not generally, if at all, hitherto known, are also recorded in the Studies on the Chyle, by M. Bouisson.* 1. When a few drops of sulphuric acid are added to the chyle of any animal, the same kind of odour is emitted as when the blood of the same animal is similarly treated: an odour, which as M. Barruel showed of the blood, and M. Couerbe of many secretions, is peculiar in each of many animals. [The fact, as M. Bouisson states, had already been observed by Vauquelin.] 2. Chyle, like blood, will often remain for a long time in its vessels without coagulating, but will coagulate rapidly on being removed from them. In one case it was fluid in a man twenty-four hours after death, but soon coagulated after its escape from the vessels. 3. The chyle-globules in the thoracic duct are, as Wagner has described them, lenticular. 4. Some experiments, apparently not very carefully performed, showed that milk injected into a dog's rectum (after purging and abstinence) was coagulated, acquired an acid reaction, and was nearly all absorbed by the lymphatics. 5. In rabbits fed for a short time with madder mixed in their food, no tinge of red is communicated to the chyle, even though the serum may be red; but if the same diet be continued till the colouring matter has thoroughly impregnated the blood, and is mixed with the urine and other secretions, it is imparted to the lymph and, thence, indirectly to the chyle.

Calculating from the analyses of Tiedemann and Gmelin, which showed a far larger proportion of fatty matter in the chyle of the recently fed, than in that of the fasting, horse, and a proportionally smaller quantity of albumen, Mr. Ross† has adduced further evidence for the view (assigned in the last Report to MM. Sandras and Bouchardat,) that the lacteals absorb none of the usual solid matters of the chyle, except the fatty matters; and that the proportion of solid matter in the chyle of the thoracic duct being less than that in the lacteal vessels is due to the chyle of the latter being diluted by mixture with the contents of the lymphatics. The other constituents of the chyle he considers to be absorbed by the roots of the portal vein, by which they are carried to the liver, and he believes that the observation of Tiedemann, respecting the apparent absence of fatty matter in the chyle when the bile-duct is tied, proves that the lacteals obtain oily matter, not from the chyle alone, but also and chiefly from the subtances secreted by the liver. He calculates from formulæ, that the bilic acid may be decomposed into an oily matter and an azotized substance which may assist to form proteine-compounds.

*Lymphatic hearts.* By Professor Stannius‡ the full discovery has been made of the existence of lymphatic hearts in birds, analogous to those in reptiles. He has found them already in the stork, ostrich, cassowary, goose, swan, diver, and hawk; and in all, with the exception of the last two, has found the walls of the heart formed by transversely striated muscular fibres. In the ostrich and cassowary these fibres form a layer from half a line to a line in thickness: in the *natatores* it cannot be discerned with the naked eye, but can (though, in some, still very sparingly) with the aid of the microscope.

It is the existence of these fibres which gives to these organs (already described as lymph-vesicles by Panizza) the right to be considered hearts. Their positions and connexions vary much in different birds. In all, several lymphatic vessels open into the cavity of the heart, and a vein proceeds from it which passes under the os ilii and joins the vena cava inferior. Lymph only has been seen in them, and they always have valves which prevent the passage of the lymph backwards into its vessels, and that of the blood from the vein into the lymphatic heart. In the swan and goose, in which alone these hearts have been observed during life, no active independent motion of their walls has yet been clearly seen, though there has been an appearance of a slow approximation of their walls, expelling their contents.

* Gazette Médicale, 1844, 29 Juin, 6 Juillet, 3 et 17 Août, &c. † Lancet, Feb. 10 and 17, 1844.

‡ Müller's Archiv, 1843, Heft v.

*Absorption by blood-vessels.** The experiments of MM. Flandin and Danger† confirm the general rule of the absorption of poisons from the digestive canal, by the branches of the vena portæ. Hence they are all found in large quantities, and some exclusively, in the liver. Their latest examinations were made on the absorption of the salts of lead, which they detected in the digestive canal, the liver, spleen, kidneys, and lungs: but not in the blood, heart, brain, muscles, or bones. Lead differs from copper in that its salts after absorption may pass off with the urine.

Experiments, by Oesterlen,‡ have also proved that mercury in its crude state is capable of being freely absorbed and circulated with the blood. It may be absorbed by the skin with the aid of friction, or from the intestinal canal. After absorption from the walls of the abdomen or the digestive canal, minute particles of it are found, for the most part, in the spleen, liver, and kidneys: and it is especially through the last two organs (at least in cats) that that which is absorbed is subsequently discharged. Globules of it have also been found in the saliva of a woman in whom it had been long applied in friction: and they existed in still greater number, (mixed, as in the saliva, with epithelium) in her urine. In one case mercury, absorbed in its metallic state, produced pneumonia with depôts of pus, apparently like that which ensues when mercury has been injected into the blood-vessels of dogs.

Mr G. Robinson,§ continuing the experiments alluded to in the last Report, and varying them, has shown that when a stream of water is made to flow through a flaccid membranous tube perforated by numerous small apertures, it will exercise a force like that of absorption upon a fluid external to the tube. Under favorable circumstances some of the fluid outside the tube is drawn into it and carried on with the current that is flowing through it. [The results of such experiments, ingenious as they are, cannot be safely admitted to prove more than that the circulation is necessary to absorption by blood-vessels, and that flaccid vessels and a rapid stream are favorable to it, by permitting imbibition and by carrying off the imbibed fluid as fast as it can enter. In the small veins and the capillaries near them the current of blood is not rapid; but, as already said, its rate is only about an inch per minute in the latter, and 1-8th faster in the former. The *drawing* power of such a current must be incalculably small; and it is, visibly, so minute that a part of the blood in every small vessel adheres to the internal surface of the wall, the adhesion between them being greater than the force of the current can overcome. It is, therefore, not imaginable that the same force should be capable of overcoming the powerful capillary attraction of the fluids held in the pores of the walls of the vessels. It is only when these fluids, by mixing with the still layer, have passed into the central current, that they are carried on with the blood.]

* I use this term rather than absorption by veins, because there is no doubt that this kind of absorption takes place through the coats of all blood-vessels that are not too thick, although, of course, the absorbed fluids are usually detected only in the veins.

† Report from the Académie des Sciences, Jan. 29, 1844, in the Gazette Méd. Fevr. 3, 1844. But in an extract from some journal in the Oesterreichische medic. Wochenschr., Mai 25, 1844, a Prof. Cozzi is said to have detected "a salt and oxyde of lead combined with the albumen of the blood of a patient suffering from lead-colic.

‡ Oest. Medic. Wochenschrift, Fevr. 24, 1844; from Roser und Wunderlich's Archiv, Heft iv, 1843.

§ Medical Gazette, June and July, 1844.

*** P. 250, last line of text, for × read +.

(*The Conclusion of this Report will appear in our next Number.*—Ed.)

